# Spatial Variability of Metal Concentrations in Seaweeds, Mussels and Surface Sediments in Gemlik Bay: an Extensive Assessment of Contamination Sources and Associated Health Risks

**DOI:** 10.1007/s12011-025-04967-6

**Published:** 2026-02-03

**Authors:** Esra Billur Balcıoğlu İlhan, Senem Çağlar, Ayşegül Mülayi̇m, Erhan Karabayır, Abdullah Aksu, Nuray Çağlar Balkıs

**Affiliations:** 1 İstanbul University Institute of Marine Sciences and Management Department of Chemical Oceanography, İstanbul, 34134 Türkiye; 2 Faculty of Science, Department of Biology, İstanbul University, Fatih, İstanbul 34134 Türkiye; 3 Turkish Energy, Nuclear and Mineral Research Agency, TR34303, K. Cekmece, Istanbul Türkiye

**Keywords:** Metal accumulation, M. galloprovincialis, U. lactuca, Macroalgae, Risk estimation, Türkiye

## Abstract

Surface sediments, mussels, and macroalgae were collected to investigate metal accumulation, distribution, pollution levels, and sources from Gemlik Bay in the southeastern Sea of Marmara. The elements Cu, Zn, V, Co, Cr, Pb, Ni, and Mn were determined in macroalgae (*Ulva lactuca* Linnaeus,1753), *(Ulva intestinalis*, Linnaeus, 1753) and mussel (*Mytilus galloprovincialis*, Lamarck, 1819) samples, while Al and Fe were also determined in sediment samples in addition to these metals. Element accumulation and metal contamination levels were determined, and health risks from mussel and macroalgae consumption were assessed across age groups. The average enrichment factor (EF) calculated for sediment samples indicates that the contamination originates from anthropogenic sources, while the contamination factor (Cf) indicates that the contamination is at a low level. Additionally, the potential ecological risk (Er) and index (PERI) reveal that, except for lead (Pb), the ecological factors for other metals are low. In addition, according to the average geochemical index (I*geo*) results, sediments were uncontaminated with Mn and Al, slightly contaminated with Fe, slightly to moderately with Cr and Pb, and moderately with Zn. Detected metals in mussels and macroalgae were assessed for health risks across age groups for the first time. Accordingly, the daily chronic intake (CDI) was calculated, and separate hazard index (HI) and target hazard quotient (THQ) values were determined for each age group. Children were identified as the most vulnerable group, highlighting the need for caution in their consumption of seafood, especially mussels and macroalgae.

**Supplementary Information:**

The online version contains supplementary material available at 10.1007/s12011-025-04967-6.

## Introduction

Although monitoring metal accumulation in marine ecosystems has been studied for many years, it remains a focus of marine pollution monitoring research today due to the continuing relevance of the issue and the bioaccumulation of metals in biota. Industrialisation, agricultural activities and population growth have made metal pollution a global problem, while increasing atmospheric pollution is causing an increase in metals like Cd, Cu, Fe, Ni, Zn and As in the oceans [36, 121, 128]. The final accumulation site of metals that do not break down and remain in the marine ecosystem after entering the water column is sediment. Therefore, monitoring of sediment clearly reflects the pollution in that area [17, 18, 125]. It is also known that metals that accumulate in sediments by collapsing from water columns are released back into the water, meaning that sediments are both a place of accumulation and a source of metal release [78, 114]. The most convenient way to monitor metal accumulation in the environment is to use benthic bioindicator organisms, which provide information about past and current pollution and accumulate pollutants in both sediments and water columns [90]. Metals in high concentrations can cause the extinction of some sensitive species and changes in community structure [25].

In recent years, the increasing levels of metals detected in sediments have become a major cause for concern [32, 69, 123, 140]. Many studies have shown that marine sediments are heavily contaminated with these metals [56, 77, 115]. Therefore, evaluating the distribution of metals in surface sediments is useful for determining pollution levels in marine ecosystem of the Sea of Marmara [8, 9, 14, 15, 104, 134, 153, 163, 180]. Organisms such as mussels, which are resistant to pollution and are good bioaccumulators due to their filter-feeding habits, are frequently used to monitor pollution [18, 64, 67, 84, 85]. The aim of this study is to determine the accumulation of metals in both surface sediments and *Mytilus galloprovincialis*, a mussel species commonly consumed by the public, in the heavily polluted Gemlik Bay, and to draw attention to metal pollution in the region and its impact on public health. Biological monitoring of seawater primarily focuses on brown algae, red algae and green algae. The uptake of metals by these algae is ranked as follows Chlorophyta > Phaeophyta > Rhodophyta [7, 131]. Therefore, green algae, particularly those of the genus *Ulva*, are widely used as bioindicator species (Akçalı and Küçüksezgin, [5]; [131].

Gemlik Bay is situated in the south-east of the Sea of Marmara and a bay with limited water movement due to the pycnocline between the surface layer characterized by low-salinity Black Sea water and the deeper bottom layer of salty Mediterranean water [23, 24]. Previous studies on metal pollution have been conducted in this region. [38] investigated the transport mechanisms of metals in surface sediments of Gemlik Bay and determined metal accumulation in lithogenic and mobile phases. They have again drawn attention to the high impact of river inputs on metal distributions in the southern shelf. [163] emphasised the effect of transport reaching the bay via rivers on the metal accumulation. In addition, [164] investigated metal accumulation in bay sediments and mussels and found that the findings in the bay were higher than in other regions of the Sea of Marmara. [86] investigated the accumulation of lead and cadmium in fish species living in the bay. Arslan-Kaya et al. [12], who examined metal accumulation in sediment samples taken from the Bay, recommended long-term monitoring of trace element concentrations in the Bay. Although a scientific study on metals was published in 2022, presenting the most recent data from 2014 for the Gemlik Bay (Arslan-Kaya et al., [12], the study was conducted only on sediment cores.

In this study, metals were not only determined in surface sediment samples, but also the contamination and sources in mussel and macroalgae samples were investigated, and contamination values were calculated and evaluated according to international indices. Additionally, risk assessments for different age groups have been calculated in the event of potential consumption of biota samples. Thus, this study represents the most recent and detailed investigation of metals—one of the most hazardous pollutant classes to the environment—in Gemlik Bay. Furthermore, by examining metal formation and the effects of local anthropogenic activities, this study will offer valuable and up-to-date insights into the sources and distribution of metal contamination in the study area. This information will be essential for formulating effective management strategies and implementing targeted measures to reduce environmental and human health risks associated with metal contamination in Gemlik Bay. Besides, the findings of this study will serve as a valuable reference for future research and contribute to addressing existing knowledge gaps in risk assessment for human health. This will enable a more comprehensive understanding of the effects of metals in Gemlik Bay, one of the most anthropogenically stressed bays in the Sea of Marmara.

## Material and Method

### Study Area

**Fig. 1:**
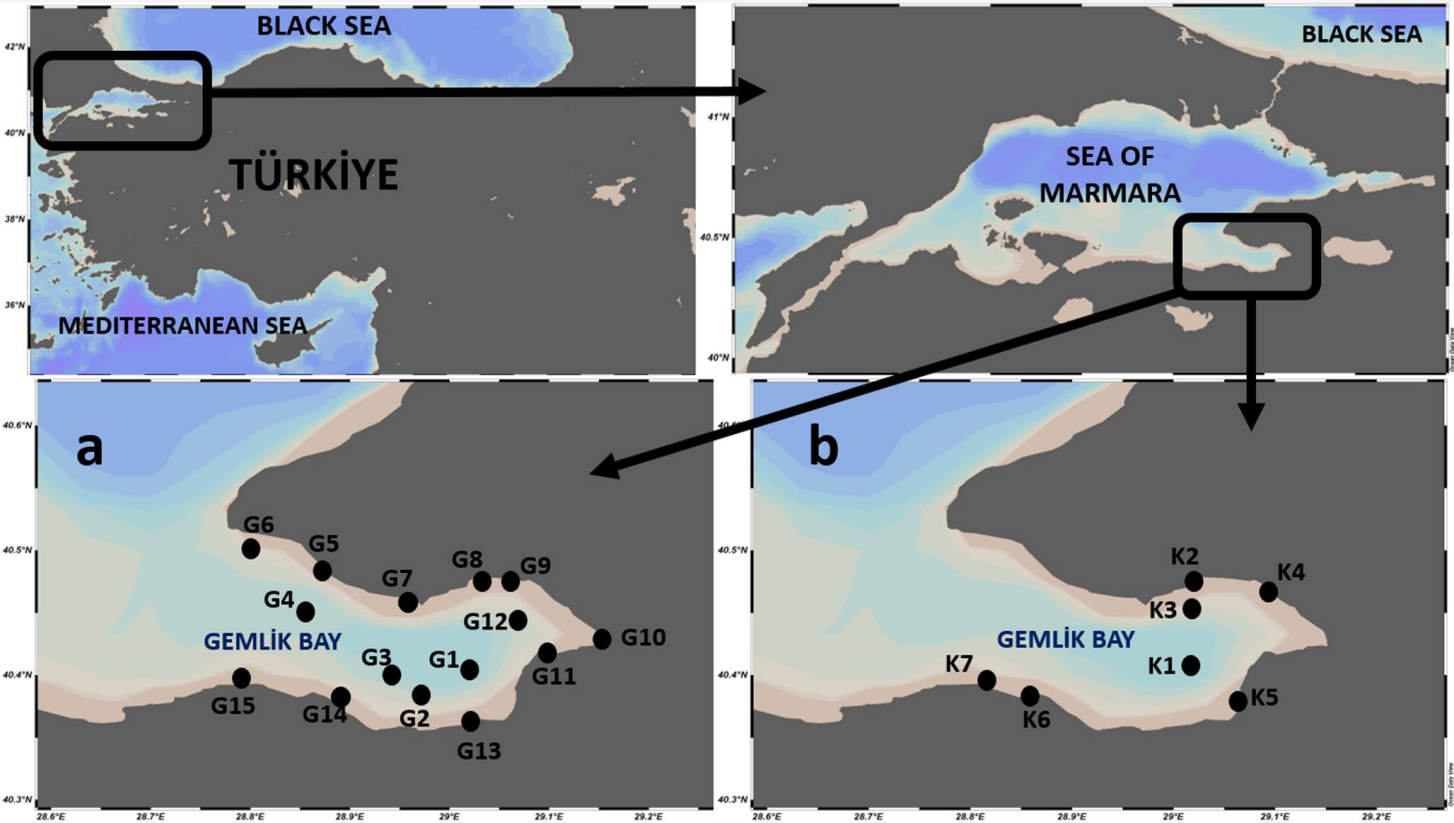
Sampling points (a- for sediments; b- for mussels and seaweeds)

The sampling study was conducted in Gemlik Bay, situated in the southeastern part of the Sea of Marmara, on 23–24 June 2021. Sediment samples were collected from 15 stations at different depths, and biota samples were collected from different locations in coastal areas. The stations where sampling was conducted are shown in Fig. 1, and the names and coordinates of the stations where biota and sediment samples were collected are listed in Table S1 and Table S2, respectively.

The study was conducted in Gemlik Bay, situated in the southeastern part of the Sea of Marmara—an inland sea of significant strategic importance [24]. The Sea of Marmara functions as a strategic marine corridor, connecting the Black Sea to the northeast via the Istanbul Strait and the Aegean Sea to the southwest through the Çanakkale Strait; due to these connections with seas of markedly different salinity levels, it features a distinctive two-layered water system [22, 24, 166] and is also an international waterway. Gemlik Bay is isolated from the rest of the Sea of Marmara by a submarine ridge approximately 50 m deep and is 36 km long and 11 km wide [177]. At 110 m deep, the Burgaz Trench in the northwest is the deepest depression within the bay [91, 177]. The main sources of income in the region are soap making, olive growing, agriculture and oil production. Due to its many ports supporting product exports, Gemlik Bay is a central point for commerce and industry [165]. The southern section of the bay hosts the majority of commercial ports in the area, among which Gemlik Port, Borusan, and Roda are the key facilities Koday and Baki [88]. Gemlik Bay is identified as one of the most severely polluted zones in the Sea of Marmara following Gulf of Izmit [163].

### Sediment and Biota Sampling Procedures

As part of the study, metal analyses were carried out on a total of 28 samples, including 15 surface sediments, 7 mussels, and 6 seaweed samples. Surface sediment samples were obtained using a Van Veen grab (0.1 m² sampling area), whereas mussel and macroalgae samples were collected from shallow coastal waters at 0–1 m depth. The mussel species used was *Mytilus galloprovincialis*, and the seaweed species used were *Ulva lactuca* and *Ulva intestinalis*. All sediment, mussel and macroalgae samples for metal analyses were analysed based on dry weight (dw). However, in order to ensure that the mussel samples were completely homogenised, they were first dried, then their moisture content was calculated and concentrations were obtained based on wet weights, and a risk assessment was carried out. Sediment, mussel and macroalgae samples were freeze-dried in a lyophiliser.

The recommended permissible metal levels in food items according to multiple organizations such as FAO/WHO, EU, CODEX has been expressed as weight-based measurements. Studies conducted by different countries and recorded based on age weight are also available. All measured metal concentrations were standardized to wet weight using the equation below, allowing for objective and comparable evaluation (Eq. 1) [41, 179]:

1 $$\:Cww=\frac{(100-Ws)}{100}\:\:x\:Cdw$$

In the equation, Cww represents the concentration of metals per wet weight unit (mg/kg); Ws represents the moisture (water) percentage (%) in the sample; and Cdw represents the metal concentration of metals per dry weight unit (mg/kg).

### Analysis Procedures

To perform metal analyses in marine sediment anf biota samples collected from Gemlik Bay, the samples were dried in a freeze dryer system and ground in a mortar. Sediment and biota samples dried using the lyophilisation method were weighed in 0.5 gram increments and placed in plastic tubes. 4 ml of nitric acid (HNO_3_), 1 ml of perchloric acid (HClO_4_) and 1 ml of hydrogen fluoride (HF) were added and the mixture was sealed. Subsequently, the dissolution process was carried out in a microwave oven (closed circuit system). After the dissolution process, 1 molar nitric acid (HNO_3_) was added to the samples and purified by completing 50 ml. The samples were then placed in plastic bottles and stored until analysis by Inductively Coupled Plasma Optical Emission Spectroscopy (ICP-OES). [96, 151, 160, 161]. Analyses were measured with a sensitivity of 0.01 µg/g. The LOQ values for metals Cu, Zn, V, Co, Cr, Pb, Ni, Mn are 0.3 µg/g, 0.1 µg/g, 0.3 µg/g, 0.1 µg/g, 0.1 µg/g, 0.6 µg/g, 0.2 µg/g and 0.03 µg/g, respectively. To calculate LOQ values ​​for each element, the standard deviation and mean of the values ​​obtained from 20 measurements of artificial seawater were calculated [110].

The metal concentrations in the prepared marine samples were measured with a Perkin Elmer Brand, Optima 7000 DV Model ICP-OES device. Calibration curves for the analyses were prepared from SCP brand standard stock solutions. The regression coefficient (R^2^) values ​​of the calibration curves are above 0.996. Three measurements were taken for each sample and the sensitivity of the analyses was at a 95% confidence interval. The accuracy of the analyses was controlled with IAEA Reference Material 405. Recovery data for the measurements are given as 97.7, 102.5, 97.3, 101, 97.4, 97.8, 96, 96.6 (%) for Cu, Zn, V, Co, Cr, Pb, Ni, Mn, respectively. The good recovery data obtained from the measurement results demonstrate the reliability of the measurement results.

### EF

The enrichment factor is calculated by chemical normalisation based on a representative element. The aim here is to identify the potential effects of metals and whether the possible sources are natural accumulation or anthropogenic activities. Aluminium is the most commonly used element for normalisation in marine sediments, as the main mineral group of fine-grained sediments is represented by aluminosilicates [72]. Aluminium is transported from land to sea as minerals from fragmented rocks, constitutes a negligible anthropogenic input, and is preserved in the marine environment. From these perspectives, Al grain size and mineralogical differences can be used for normalisation [20, 40, 72, 184, 185]. In this study, Al was selected for grain size normalisation (Eq. 2).

2 $$EF=\;\lbrack(CM/CAl)sample\rbrack/\lbrack CM/CAl)reference\rbrack$$

Here, C_M_ stands for the metal content in the sediment (ppm or mg/kg); C_Al_ stands for the Al content at the same point (ppm); C_M−reference_ represents the reference concentration of the measured element; and C_Al−reference_ represents the reference concentration of the Al element.

The EF value indicates different results according to the scales used in the assessments. These are divided into two categories: origin (natural-mineralogical and anthropogenic) and degree of contamination. If the EF value is < 1.5, the origin is natural; if EF > 1.5, the origin is anthropogenic [174].

### I_geo_

Like enrichment factors (EF), the geo-accumulation index is a reference established by [105] used to determine whether metal concentrations are at levels that cause pollution. When calculating this reference, Background values employed for the EF calculation were taken as baseline reference values (Eq. 3).

3 $$I_{geo}\:=\:\log_2\;\lbrack C_n/1.5\;x\;B_{bg})\rbrack$$

C_n_ represents the element concentration measured in sediment samples, B_bg_ represents the geochemical background value of metal concentration, and 1.5: Bacground matrix correlation factor (due to lithogenic effects). Values between 0 and 5 indicate pollution ranging from minimal to extreme [105]. Accordingly, 0 < Igeo < 1: slightly contaminated; 1 < Igeo < 2: moderately contaminated; 2 < Igeo < 3: contaminated; 3 < Igeo < 4: significantly contaminated; 4 < Igeo < 5: heavily contaminated; 5 < Igeo: extremely contaminated sediments [105] Barbieri, 2016).

### Cf and PLI

One of the calculations used to determine pollution levels in sediment samples is the contamination factor (Cf). [68] pollution factor is determined by comparing the metal content in the sample with the same metal value in the background environment (Eq. 4).

4 $$Cf\:=\:Cs/Cr$$

Here, Cs refers to the metal concentration in the sediment sample, and Cr to the concentration in the reference sample. Reference values are taken from Krauskoff [89]. According to this, Cf ≤ 1: low; 1 ≤ Cf ≤ 3: medium; 3 ≤ Cf ≤ 6: significant; 6 ≤ Cf: sediments with very high levels of contamination.

The PLI serves as an easy method for assessing and comparing the cumulative metal pollution at a designated sampling location, calculated from the Cf values as shown below (Eq. 5).

5 $$PLI\;=\;{(Cf_1\;x\;Cf_2\;x\;Cf_3\;x\dots\dots\;Cf_n)}^{1/n}$$

Cf is the contamination factor and n is the number of metals measured. PLI ≤ 1 means no pollution, 1 ≤ PLI ≤ 2 means moderate pollution, 2 ≤ PLI ≤ 3 means high pollution, and 3 ≤ PLI means very high pollution [154].

### Er and PERI

The ecological risk (Er) value of a specific metal is available (Eq. 6). Parameters developed by [68] are used to determine the potential risk index of metals found in sediment samples (Eq. 7).

6 $$Er\:=\:Tr\;x\;Cf\;$$

7 $$PERI=\sum_{i=1}^n\;ER$$

Tr refers to the coefficients given by [68] as the ”toxic response factor” for Zn, Cr, Pb, Ni, Co and Cd, which are 1, 2, 5, 5, 5 and 30 respectively. According to the results obtained, Er values are evaluated in 5 categories and PERI values in 4 categories. Er < 20, PERI < 30: low risk; 20 ≤ Er ≤ 40, 30 < PERI < 60: moderate risk; 40 ≤ Er ≤ 80, 60 < PERI < 120: significant risk; 80 ≤ Er ≤ 160, PERI > 120: high risk; Er > 160: very high risk.

### Health Risk Assessment

Metal concentrations in mussels and macroalgae samples were used to calculate oral exposure levels for three different age groups: children, adolescents, adult women and adult men. Four different age categories (children, adolescents, adult women, adult men) Average body weights applied were 21 kg for children aged 6, 51 kg for 14-year-old adolescents [170], 75 kg for female adults (mean age 79.1 years), and 84 kg for male adults (mean age 73 years) WorldData, [173]. The annual mussel consumption rate is given as 1.19 kg/year, as recommended by EUMOFA [53]. Since there is no specific regulation regarding the consumption amount of macroalgae, it has been considered at the same rate as mussels. Using the following equation, the CDI of each metal per mussel meal was determined for the required age groups (Eq. 8).

8 $$\:CDI=\frac{\mathrm{C}\:\mathrm{x}\:\mathrm{E}\mathrm{F}\:\mathrm{x}\:\mathrm{E}\mathrm{D}\:\mathrm{x}\:\mathrm{I}\mathrm{R}}{\mathrm{A}\mathrm{T}\:\mathrm{x}\:\mathrm{L}\mathrm{T}\:\mathrm{x}\:\mathrm{B}\mathrm{W}}$$

C: Metal concentration (mg/kg ww), EF: exposure frequency (365 days/year), ED means exposure duration (life expectancy: 73 years for men, 79 years for women, 6 years for children, and 14 years for adolescents); IR refers to the ingestion rate, representing the seafood consumption rate (mg/person/day); AT denotes the averaging time for non-carcinogenic effects (365 × ED days); LT indicates the lifetime or exposure duration (in years); BW stands for body weight, specific to Turkish men, women, children, and adolescents.

THQ (Target hazard quotients) values for each metal were calculated using the following equation (Eq. 9) to assess non-carcinogenic risks to human health.

9 $$THQ\:=\:CDI/RfD$$

If THQ < 1, it is unlikely that exposure to metals will result in significant adverse effects (USEPA [167]. If THQ ≥ 1, this indicates that health is seriously threatened and appropriate measures and protective precautions should be taken. The HI (total hazard index) obtained by adding these THQ values were also applied in assessing potential non-carcinogenic health risks from multiple metal contaminants (Eq. 10).

10 $$\:HI={\textstyle\sum_{i=1}^n}\;THQ$$

### Total Organic Carbon (TOC) and CaCO_3_

The total organic carbon content (C*org*) in surface sediment is calculated using the Walkey-Blake method [61, 96]. After drying, each sample was weighed to 0.5 g prior to further processing, reacted with 10 ml of 1 N potassium dichromate (K₂Cr₂O₇) and 20 ml of concentrated sulfuric acid (H₂SO₄), and then diluted to 200 ml. 10 ml of concentrated phosphoric acid (H_3_PO₄), 0.2 g of sodium fluoride (NaF), and 1 ml of diphenylamine were added, and titration with iron ammonium was performed. After applying the same procedure to glucose and blank samples, calculations were made according to the following formula.

11 $$\%Corg\:=\:3,951/g\;x\;(1-T/S)$$

Here, g: weight of the dried sample; S: iron ammonium solution, for blank (ml); T: iron ammonium solution, for sample (ml).

For the analysis of calcium carbonate in sediment, 1 g of dry sample was reacted with 10 ml of 1 molar hydrochloric acid (HCl). The resulting CO_2_ gas was measured volumetrically, and the percentage of carbonate was calculated according to the Eq. 12 [96].

12 $$\%\;CaCO3\:=\:100\;x\;V/W$$

In the formula, V represents the amount of CO_2_ gas released (cm), and W represents the amount of CaCO_3_ equivalent to 1 gram.

### Bioaccumulation Factor (BSAF)

BSAF was calculated using the equation below to assess how effectively seaweed or algae can accumulate elements from their surrounding environment, such as sediment or seawater (Eq. 13):

13 $$BSAF\:=\:C_{algae}/C_{sediment}\;or\;C_{algae}/C_{seawater}$$

14 $$BSAF\:=\:C_{algae}/C_{sediment}\;or\;C_{algae}/C_{seawater}$$

Here, C_algae_ = element values in algal tissues (mg/kg), and C_sediment_ and C_seawater_ are element values in sediment and seawater, respectively (mg/L). Since there were no metal data related to water in this study, calculations were made using data related to sediment.

### Data Analysis and Statistics

Prior to performing Principal Component Analysis (PCA), the normality of the data was assessed using both the Shapiro–Wilk and Kolmogorov–Smirnov tests. Since the majority of the metal concentration data did not follow a normal distribution (*p* < 0.05), a log₁₀-transformation was applied. PCA was conducted using SPSS 30.0.0 and PAST 5.0.2 to investigate the correlations between metal concentrations in mussel and algae samples collected from 7 stations in Gemlik Bay, surface sediment metal concentrations from 15 stations, as well as calcium carbonate (CaCO_3_), total organic carbon (TOC) and station depth. Prior to this analysis, the linear correlation between the variables was determined by means of the Spearman rank correlation coefficient (*p*).

## Results and Discussion

### Seaweed Contamination

Metal levels were examined in macroalgae samples collected from 5 of the 6 stations where macroalgae samples were collected. The *U. lactuca* species was found in 5 stations, and the *U. intestinalis* species was found in one station. Copper (Cu), zinc (Zn), vanadium (V), cobalt (Co), chromium (Cr), lead (Pb), nickel (Ni) and manganese (Mn) were determined in the collected macroalgae samples. Metal concentrations across stations and specifically in *U. lactuca* ranged from 3.78 to 890.7 mg/kg (dw). The lowest value was detected in Co metal, while the highest was detected in Mn metal. The ranking of other metals determined in macroalgae samples was found to be Mn > Zn > Cr > Ni > V > Cu > Pb > Co (Table 1).

**Table 1:** Metal concentrations in seaweed (*Ulva lactuca*- K4: *Ulva intestinalis*) samples (mg/kg - dw)

		mg/kg							
	Stations	Cu	Zn	V	Co	Cr	Pb	Ni	Mn
K1	Armutlu	27.81	32.777	11.124	< 0.1	10.131	< 0.6	15.495	150.179
K2	Narlı	14.77	19.764	< 0.3	< 0.1	< 0.1	< 0.6	< 0.2	25.155
K3	Kapaklı	18.10	60.275	11.737	3.780	11.538	< 0.6	19.296	97.076
K4	Gemlik Port	16.71	73.418	9.351	< 0.1	13.132	< 0.6	19.698	278.552
K5	Kurşunlu Pier	84.49	330.020	212.724	40.358	310.139	62.712	214.712	890.656
K7	Kumyaka	43.58	43.781	14.129	< 0.1	6.567	< 0.6	9.950	62.488
	Min	14.773	19.764	9.351	3.780	6.567	62.712	9.950	25.155
	Max	84.493	330.020	212.724	40.358	310.139	62.712	214.712	890.656

*Ulva* sp. has a wide distribution and possesses various ecological characteristics that make it a valid indicator, including sensitivity to certain natural and human-induced impacts and the measurability of species responses to these impacts [106]. However, comparative studies on trace element bio-tracking in vascular plants and algae are still quite limited in marine environments [39].

Various bioactive compounds produced by green algae (macroalgae/seaweed) are widely used in agriculture as biofertilizers, in animal nutrition, and as food for human consumption [76]. Macroalgae are considered valuable biomonitors because they are stationary, abundant, easy to identify, long-lived, readily available, and efficient in accumulating metals [142]. Millions of people around the world rely on green macroalgae and green seaweed as an important component of their diet [142]. Green macroalgae contain a rich composition of nutrients and bioactive compounds, including lipids, carbohydrates, proteins and ash. They are also a source of amino acids, plant hormones such as abscisic acid, gibberellins, cytokinins and auxins, as well as antimicrobial agents and essential elements like potassium, phosphorus and nitrogen [130]. Therefore, risk assessments have been conducted for potential use cases in this study as well.

The metals ranked from high to low in our study are similar to other studies in terms of both their high values and their rankings [57, 62, 101]. A comparison of metal accumulation in *Ulva* sp. samples from different regions is provided in Table 2. Previous studies have shown that metal concentrations in seagrasses and macroalgae significantly reflect the concentrations of these elements in bottom sediments [100, 168].

**Table 2:** Comparison of metal contents in seaweed samples from various regions (mg/kg- dw)

Location	Cu	Ni	Pb	Zn	Co	Cr	Mn	Reference
Ras Beirut, Lebanon	52.5	43.1	37.5	206	-	16.3	-	[143]
Sicilian Coast, Italy	2.35	-	1.52	14.6	-	0.46	-	[31]
Tyrrhenian Sea Central Italy	6.4	-	2.28	-	-	2.06	-	[38]
Egyptian Red Sea	8.3	-	37.20	40.7	-	-	16.9	[1]
Assateague Island, Maryland	0.7	-	-	6.9	-	-	22.4	[35]
Chinoteague Island, Virginia	0.7	-	-	13	-	-	50.5	[35]
El Mea Bay (Egypt)	7.24	-	-	27.4	6.6	-	33.3	[2]
Eastern Harbour (Egypt)	-	-	-	63.1	7.1	-	73.9	[2]
Black Sea Türkiye	3.9	-	0.648	22.4	-	-	19.1	[157]
Manastir (Tunusia)	8	-	12.6	68	-	-	13	[176]
Çanakkale, Türkiye	8.5	-	0.0056	50.1	-	-	-	Akçalı and Küçüksezgin [5]
İzmir, Türkiye	14	-	0.0175	67.8	-	-	-	Akçalı and Küçüksezgin [5
Jijel, Algeria	2.37	-	1.001	5.4	-	-	-	[92]
Msimobazi, Tanzania	15.8	-	-	17.3	-	-	40.8	[109]
Vadinar, India	16	-	1	326	-	-	7.8	[34]
The Sea of Marmara	18.32	-	19.32	41.3	-	-	-	Özyiğit et al., [113]
Lebanese Coast	5.64	3.99	4.44	8.9	1.3	3.74	128	[62]
Gemlik Bay	84.5	214	62.6	330	40,4	310.1	890.7	Present study

In this study, metal determination was also performed on the macroalgae species *U. intestinalis* found at station K4. [28] have indicated that *U. intestinalis* is a multifaceted indicator of dissolved metals. Researchers have mentioned the advantage of *U. intestinalis* in reflecting changes in metal levels in the environment more quickly. [136] also recommend the use of *U. intestinalis* as an indicator of metal pollution in estuaries and coastal areas.

Particularly, *Ulva lactuca* shows a pronounced tendency to accumulate trace metals such as Mn, Cu, Zn, Pb, and Cd [71]. Studies on *U. lactuca* have generally reported high Fe levels, which are predominant in Chlorophyta [2]. When compared to other studies conducted in the Sea of Marmara, Mediterranean Sea and Black Sea (Table 2), trace element levels in *Ulva sp* macroalgae samples in our study are generally higher.

Since the introduction of a regulation limiting leaded gasoline consumption in 1970, there has been a reported downward trend in Pb concentrations in the aquatic environment [149]. Compared to the 1977 study in Beirut, Pb levels in *Ulva lactuca* have diminished over time [143]. It is important to consider the disparity between the detection methods used in the past and the modern techniques currently applied. Furthermore, it should be note that industrialization and urbanization are also developing rapidly and, as a result, anthropogenic sources entering the marine environment are diversifying and becoming more varied. [87] highlighted that elevated Pb exposure in children can result in significant toxicity, including anemia, weakness, and impairment of kidney and brain functions. Pb values determined in different regions of *U. lactuca* species, in the Red Sea of Egypt [1], along the coast of Lebanon [62], in Ras Beirut, Lebanon [143], and in Manastir, Tunisia [176] are lower than the values detected in the Gemlik Bay in our study. Similarly, our findings in Zn concentrations are also higher than Zn findings in various regions.

Metal pollution in the marine environment, whether from natural or human sources, poses serious threats due to its toxicity and cumulative properties [65, 144]. In addition, Cu, Pb, Mn, Se, Ni, Pb, and Zn remain in the environment for a long time because they cannot be broken down or destroyed [141]. Metals also include trace amounts of essential elements such as Fe, Ni, Zn, and Mn, which are vital for the normal physiological processes of organisms. Conversely, toxic or non-essential elements like Cr and Pb pose health risks even at trace levels [159, 175]. These elements are associated with a range of acute and chronic diseases, including high blood pressure, liver and kidney damage, nervous system impairments, anemia, cognitive disorders, cancer, and mood disturbances [99, 103].

The presence of anthropogenic pollution is indicated by high concentrations of Mn in macroalgae samples. Except for Mn values found in Chincatague Island [35] and East Port of Egypt [2], studies conducted in other regions are lower than the Mn findings in our study. Generally, as demonstrated by most studies, *U. lactuca* has been shown to be a good candidate for biomonitoring studies Özyiğit et al., [113].

#### CDI, EWI, THQ and HI

**Fig. 2:**
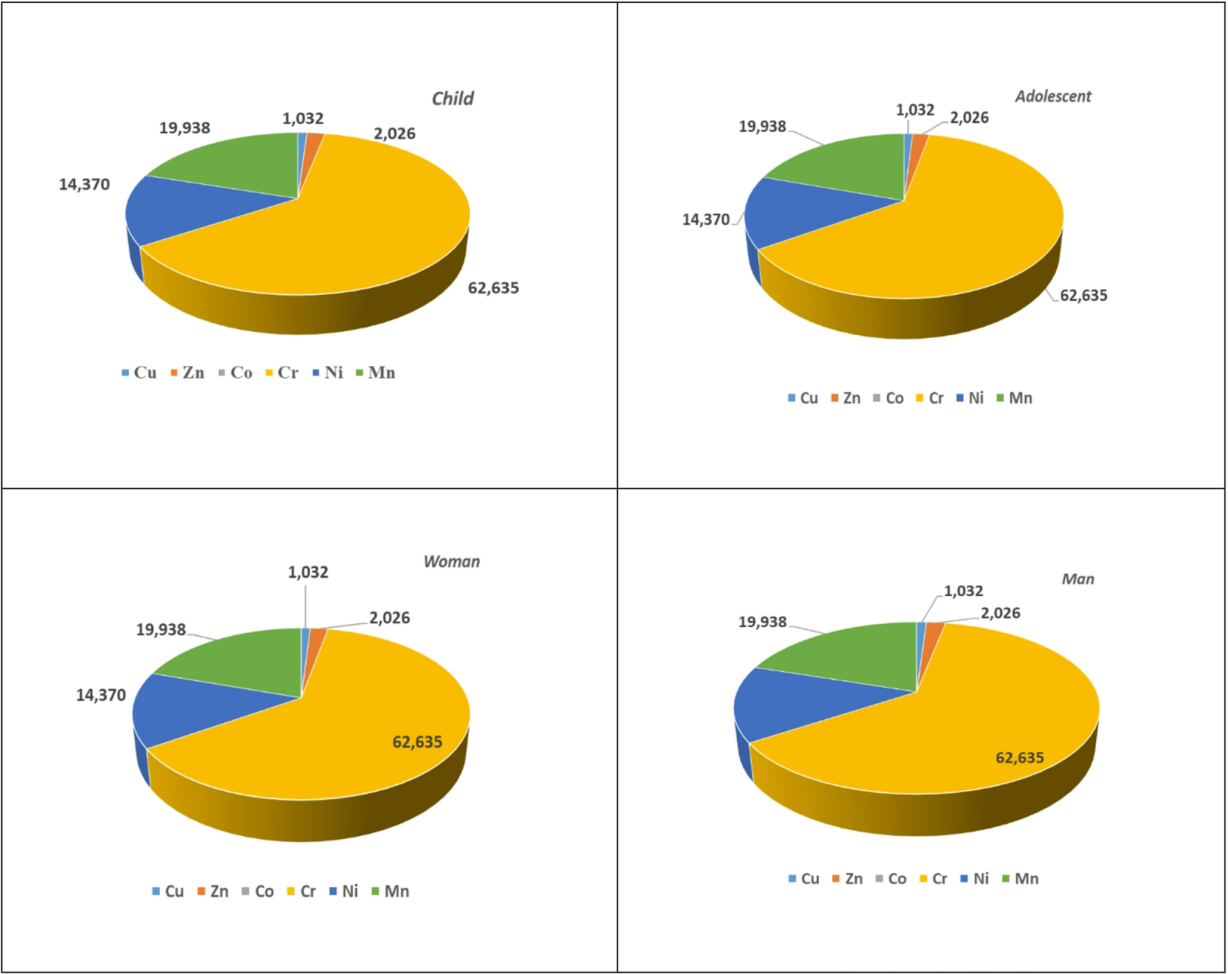
THQ percentage of various risk groups for seaweeds (*Ulva sp*)

Although macroalgae samples are not directly consumed like fish or mussels, they are used in the ingredients of various food supplements. Therefore, CDI values have been calculated using metal concentrations to assess potential health risks in case of potential use (Table S3). Based on these values, estimated weekly intake (EWI) have also been calculated (Table S4). CDI values in children across all stations and metals ranged from 1.86E-01 (Co) to 4.38E + 01 (Mn). CDI values ranged from 5.38E-02 (Co) to 1.26E + 01 (Mn) for adolescent groups, 6.69E-03 (Co) to 1.58E + 00 (Mn) for female adult groups, and 9.51E-03 (Co)- 2.24E + 00 (Mn). When viewed through these values, the highest CDI value was found in the child risk group. This situation indicates that children are more exposed than other risk groups. The THQ and HI values of metals for all age groups that could potentially consume algae are shown in Table S5, and the THQ % graphs for all risk groups are shown in Fig. 2.

According to the risk assessment of macroalgae at Gemlik port (K4) and Kurşunlu station (K5), THQ and HI values were found to be significantly high. This situation is related to the fact that these stations are exposed to both ship traffic and discharges, as reported by [182]. In most macroalgae, THQ and HI > 1 have been observed, indicating a significant non-carcinogenic effect, and in this case, appropriate precautions and protective measures should be taken.

Among all age groups, children are the most susceptible to the harmful effects of metal exposure. The THQ and HI values in our study were higher than the HQ and HI values for green algae reported by [57].

### Mussel Contamination

The elements Cu, Zn, V, Co, Cr, Pb, Ni and Mn were determined in the collected mussel samples. Metals detected in samples of the mussel species *M. galloprovincialis* were found in the range of 3.79–1639.34 mg/kg. The lowest value was found in Co at the Kurşunlu pier (K5) station, while the highest value was found in Zn at the Armutlu (K1) station. The concentrations of metals, based on their average concentrations, are ranked as follows: Zn > Cu > Mn > Ni > Cr > Co. Neither V nor Pb elements were detected in any station in the mussel samples. The findings are presented in Table 3.

**Table 3:** Metal concentrations in mussel (*Mytilus galloprovincialis*) samples (mg/kg - dw)

		Metals (mg/kg)							
Stations		Cu	Zn	V	Co	Cr	Pb	Ni	Mn
K1	Armutlu	23.77	1639.34	< 0.3	4.75	8.2	< 0.6	12,13	41,97
K2	Narlı	90.23	332.56	< 0.3	< 0.1	< 0.1	< 0.6	< 0.2	12.09
K3	Kapaklı	10.67	603.33	< 0.3	< 0.1	6.67	< 0.6	7.67	98.33
K4	Gemlik Port	14.32	734.09	< 0.3	< 0.1	6.36	< 0.6	10.68	32.27
K5	Kurşunlu Pier	20.17	1010.35	< 0.3	3.79	10.17	< 0.6	12.41	43.97
K6	Mudanya	9.55	265.67	< 0.3	< 0.1	< 0.1	< 0.6	5.97	14.78
K7	Kumyaka	124.31	750.98	< 0.3	< 0.1	5.69	< 0.6	10.20	17.26
	Min	9.55	265.67	-	3.79	5.69	-	5.97	12.09
	Max	124.31	1639.34	-	4.75	10.17	-	12.41	98.33

Metal concentrations determined in mussel samples collected from seven different stations are given as dry weight (dw) for easier comparison with studies in the literature. However, consumption-based risk assessments have been calculated based on body weight (ww). The Zn levels identified in present study were considerably higher than the findings of Germany’s, and the Apulian coast of Italy [148], and the permitted limit of 200–500 mg/kg set by the United States Environmental Protection Agency USEPA [52]. The Food and Agriculture Organization (FAO) Collette and Nauen, [37] has established 1000 mg/kg as the maximum permissible zinc concentration in molluscs.

According to the EU Commission Regulation, the maximum value given for Pb is 1.50 mg/kg (European Commission [54]. In this study, however, as the Pb values at all stations remained below the measurement limits, no comparison could be made in terms of Pb.

As a fundamental element, Cu is easily taken up by aquatic organisms, reflecting its comparatively elevated levels in such seafood [181]. This element is essential for the proper functioning of the immune, hematopoietic, and cardiovascular systems, and it also helps control oxidative stress [138]. Excessive Cu exposure is associated with gastrointestinal issues and can impair the liver, immune system, neurological functions, and reproductive health. The average Cu element concentration found in this study is 124.31 mg/kg (dw), which is much higher than the values found in other regions, as shown in Table 4. On one hand, the Cu levels in this study exceed the limits permitted by the FAO (Collette and Nauen [37]at 70 mg/kg, the United Nations Environment Programme [162] at 20 mg/kg, and Türkiye [152]. USEPA [52] sets the maximum allowable Cu concentration in molluscs between 50 and 150 mg/kg; the Cu levels measured in this study comply with these standards.

**Table 4:** Comparison of metal contents in mussel samples from various regions (mg/kg- dw)

Location	Co	Cr	Cu	Zn	Ni	Pb	Reference
Norwegian Sea	2.7	1.6	7.6	97.4	3.5	0.5	[117]
Baltic Sea (Norway)	8.3	1.6	12.1	4.9	9.6	4.9	[117]
Northern Sea of Marmara	1.9	3.5	6.7	319.9	5.3	< dl	Topçuoğlu et al. [155]
İstanbul Strait	-	19.3	58.2	462.3	43.7	25.4	Aksu [6]
Western Scheldt estuary (Netherlands)	-	0.8	9.5	83.1	0.63	2	[102]
Moraccan Atlantic Coast	-	-	43.1	37	-	26.5	[97]
Balearic Islands	-	0.5	4.8	234.2	-	2.5	[45]
Brown Bay, Argentina	-	-	2	130.7	-	-	[63]
Montenegro Coasts (Adriatic Sea)	3.92	-	-	-	3.35	9.10	Joksimovic et al. [80]
Galicia and Cantabria, Spain	-	-	10.1	470	-	26.6	[21]
Saronikos Gulf, Greece	-	3.7	20	269	4.9	-	[150]
Apulian Coast (Italy)	-	3.1	8.2	75.3	-	1,5	[148]
Chacopata- Bocaripo Lagoon (Venezuela)	1.7	-	9	-	8.5	0.2	[119]
Island of Sylt, Germany	0.8	1	8.2	2	2	1.2	[70]
Tekirdağ, Sea of Marmara	-	2.5	3.5	89	2.5	9.2	Dökmeci [48]
Cala Iris Sea	< dl	2.9	5.8	162	3.1	< dl	[13]
South Africa (Saldanha Bay)	-	0.38	0.35	37.4	-	0.8	[60]
Asturias coast North of Spain	-	1.5	-	291	3.3	9.6	Sanz- Prada et al. [132]
Gemlik Bay	4.8	10.2	124.3	1639	12.4	< 0.6	Present study

Cr concentrations in mussel tissues were found to range from 0.5 to 19.3 mg/kg in the western Mediterranean Balearic Islands [45] and Istanbul Strait Aksu [6] mussels (Table 4). No maximum permissible levels for Cr in fish or mollusks are specified by the USEPA [52], EFSA [55], or [162]. The USFDA (1993) sets the maximum chromium content in mollusks at 12 mg/kg, whereas the FAO (Collette and Nauen, [37] allows a maximum of 1 mg/kg. The highest Cr level of 10.2 mg/kg found in mussel tissues taken from Gemlik Bay was much higher than the limits permitted for fishery products by the FAO (Collette and Nauen [37].

#### CDI, EWI, THQ and HI

Metal concentrations in mussels were used to assess potential consumption levels and risks for four different age groups: Aduls (women and men), adolescents and children.

**Fig. 3:**
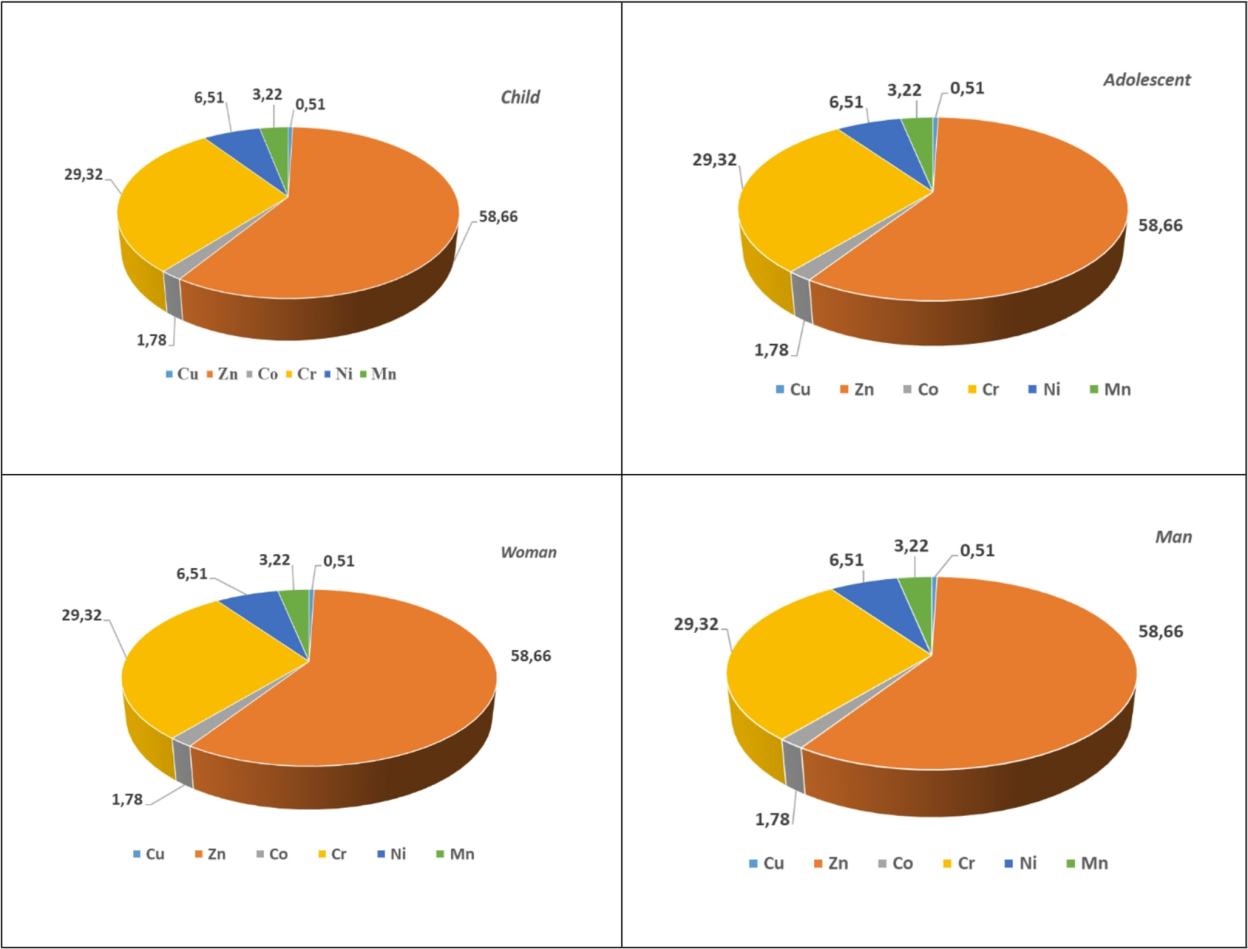
THQ percentage of various risk groups for mussels (*Mytilus galloprovincialis*)

CDI values have been calculated for health risks using the metal content of mussels. CDI values in children across all stations and metals ranged from 2.45E-02 (Co) to 1.06E + 01 (Mn). CDI values for adolescent groups are 7.04E-03 (Co) − 3.04E + 00 (Mn), for adult female groups, 8.82E-03 (Co) − 3.81E-01 (Mn), and for adult male groups, 1.25E-03 (Co) − 5.42E-01 (Mn) (Table S6). When evaluated based on these values, the highest CDI value was found in the child risk group. Similarly, EWI values are also highest in the child risk group (Table S7). This situation indicates that children are more exposed than other risk groups. THQ values for metals in all age groups consuming mussels are presented in Table S8 and THQ % graphs for all risk groups are shown in Fig. 3. THQ and HI > 1 were found in all Gemlik mussels, indicating a non-carcinogenic but monitorable situation for all risk groups. The highest risk is observed in children, who are the most vulnerable group for exposure to metals. This result is similar to that reported by [147], who indicated that green mussels collected from various locations on the east coast of Java had HQ values higher than 1, and by [120], who reported HQ values above 1 in green mussels collected in Cilincing (Indonesia).

In the mussels collected within the scope of this study, as well as in the study conducted by [120] Pb levels remained below the detection limit, therefore risk analysis could not be performed.

In this study, the stations with the highest metal concentrations in mussels were found to be Armutlu (K1), Kurşunlu (K5), Kapaklı (K3), and Gemlik Port (K4). Among these stations, Kurşunlu, Armutlu, and Gemlik Port stations have both high population density and discharge. Pollution effects are greater in areas close to residential areas and in areas with port or discharges of sewage compared to other areas [171]. [182] reported high pollution index values in Kurşınlu, Narlı and Gemlik, where ship traffic is very heavy. Researchers have also mentioned pollution from industry and agriculture in addition to vessel traffic in Gemlik and Kurşunlu.

### Sediment Contamination

In the collected surface sediment samples, in addition to Cu, Zn, V, Co, Cr, Pb, Ni, and Mn, Aluminium (Al) and Iron (Fe) were also determined, and the results are presented in Table 5. The assessment of whether the values were high or normal [89] was also carried out using the reference values specified for each metal. Additionally, the necessary pollution indices, risk assessments, and statistical analyses have been conducted.

**Table 5:** Metal concentrations in surface sediments (mg/kg-dw)

		mg/kg								(%)	
Stations		Cu	Zn	V	Co	Cr	Pb	Ni	Mn	Al (%)	Fe (%)
1	G 1	101.912	358.762	248.374	42.973	362.705	81.214	248.374	1166.96	12.34	8.36
2	G 2	84.997	290.571	191.737	33.999	300.455	63.847	199.644	826.250	2.63	6.42
3	G 3	86.592	221.468	201.516	29.928	205.507	33.320	123.304	736.233	6.46	6.90
4	G 4	19.949	77.819	49.180	8.572	83.152	13.826	53.131	225.163	2.86	1.84
5	G 5	65.057	221.513	165.835	34.724	287.368	59.669	209.539	778.288	6.65	6.67
6	G 6	36.216	145.458	81.932	17.237	129.824	47.892	74.807	653.077	4.1	3.01
7	G 7	67.637	283.130	192.096	28.510	141.172	93.197	42.470	1136.453	10.11	7.2
8	G 8	56.175	163.745	237.052	38.247	137.849	23.904	46.016	1145.418	4.96	8.37
9	G 9	121.458	299.188	285.318	42.996	273,430	40,816	148.801	947.097	14.70	10.56
10	G 10	124.94	366.79	380.40	53.92	290.35	63.15	168.61	989.13	15.30	12.4
11	G 11	68.767	303.210	178.359	31.114	245.739	53.508	164.487	913.595	6.78	6.88
12	G12	49.105	147.515	141,153	26.839	188.270	19.085	137.773	652.087	7.79	5.31
13	G 13	34.055	154.724	176.181	27.165	144.685	29.921	81.102	913.386	6.34	6.18
14	G 14	71.400	267.506	139.260	27.931	578.285	46.814	128.639	668.765	1.71	4.56
15	G 15	70.255	233.525	148.031	33.248	344,350	51.652	225.609	641.203	5.88	6.06
Min		19.9	77.8	49.2	8.6	83.2	13.8	42.5	225.2	1.71	1.84
Max		121.5	358.8	285.3	43.0	578.3	93,2	248,4	1167,0	14.70	10.56
Mean		67.9	229.0	174.4	31.0	242.9	46.8	136.2	822.9	7.18	6.3
Non-polluted		< 25	< 90		-	< 25	< 40	< 20	Sediment Quality Guidelines (SQGs) (Smith et al.,[146]; [98] ANZECC and ARMCANZ [10]		
Moderately polluted		25–50	90–200		-	25–75	40–60	20–50			
Highly polluted		> 50	> 200		-	> 75	> 60	> 50			
ISQG- Low		65	200		-	80	50	21			
ISQG- High		270	410		-	370	220	52			
Canadian ISQGs		18.7	124	-	-	52.3	30.2	-	CCME [33]		
PEL (Probable Effect Level)		197	315	-	-	90	112	36			

**Fig. 4:**
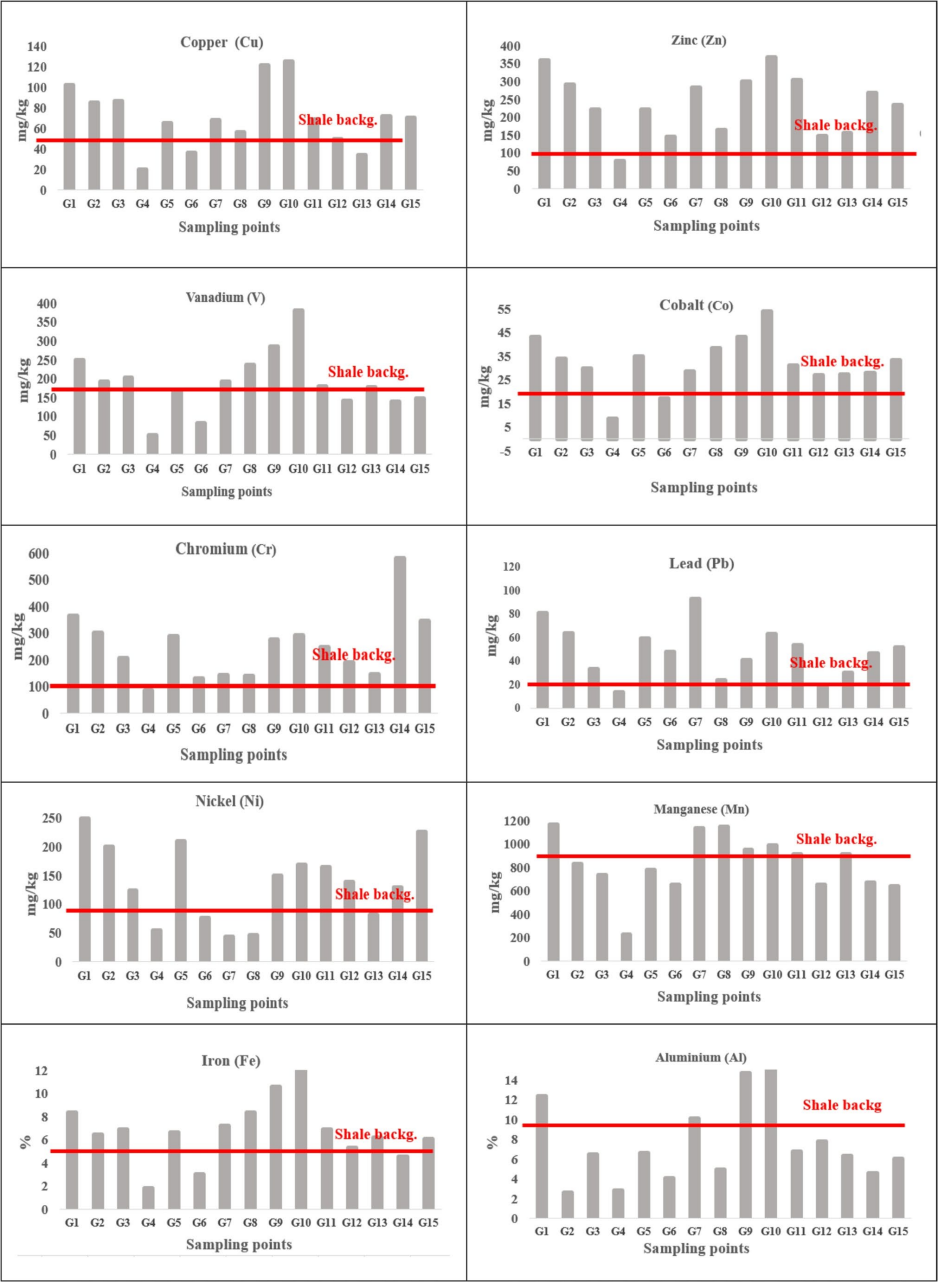
Comparison of element values in surface sediments of Gemlik Bay with shale values

No metals were detected below the measurement limit in any surface sediment samples, and all metals studied were detected at all stations. Accordingly, the Cu has the lowest and highest values ranging from 19.9 to 124.9 mg/kg, with an average value of 70.6 mg/kg. Cu is an abundant element in nature and ranks twenty-eighth among the metals found in the Earth’s crust [93]. It can enter the aquatic ecosystem through atmospheric deposition or terrestrial transport, and is soluble in water after entering the environment. Cu also enters the environment anthropogenically through fungicides, pesticides, wastewater, fossil fuels, mining and industrial processes [169]. In this study, Cu values in surface sediments at all stations except three (G4, G6, G13) were found to be above the shale values (50 mg/kg) specified by Krauskopf, indicating Cu-related pollution at most stations (Fig. 4).

Across all stations, Zn concentrations in surface sediments ranged from 77.8 to 358.8 mg/kg, with an average value of 229 ± 74.5 mg/kg. Zn is a highly prevalent environmental pollutant [43, 58]. Domestic wastewater, fossil fuels, metal industry products, cement products and atmospheric deposition are the main sources of pollution entering the aquatic environment [44]. Zn contents in the Bay were found to be above the shale average (90 mg/kg) except at the G4 (Fıstıklı Offshore) station. The intervals and values found were higher than those reported by [116] in the Kocasu Delta (Sea of Marmara), and similarly, the average Zn values were above the shale value. This indicates enrichment with Zn.

V values in surface sediments ranged from 49.2 to 285.3 mg/kg across all stations, with an average of 174.4 ± 58 mg/kg. V is a transition metal found in the Earth’s crust that is resistant to corrosion and has oxidation values ranging from 0 to + 5. Its distribution in the environment is caused by the burning of fossil fuels, waste water, the metal industry that processes vanadium, and atmospheric deposition carried by the wind. Furthermore, oil ash waste also enters the aquatic environment through seawater [27]. Since V values are above the reference value (130 mg/kg) at most stations, there is also enrichment in terms of V (Fig. 4).

Co values were found to be between 8.6 and 43 ppm, with an average of 31 ± 9 mg/kg. Co is dispersed into the environment through natural atmospheric deposits, soil, seawater, volcanic eruptions, and forest fires [108]. These are introduced into the ecosystem through Human-related activities like mining, phosphate fertilizer application, the disposal of cobalt-containing waste, fossil fuels, exhaust fumes and industrial metal processing (Smith and Carson, [145]. Since Co values remained below shale values (20 mg/kg) at only two stations (G4 and G6) in the bay, enrichment can be observed at all other stations (Fig. 4).

Cr values in surface sediments in the study ranged from 83.2 to 578.3 mg/kg. The average Cr was found to be 242.9 ± 120.4 mg/kg. Only the Cr value in G4 remained below the shale value (100 mg/kg), while it was higher than the shale value at other stations, suggesting that most stations were somewhat enriched in Cr. Cr is used anthropogenically in the steel and metal industry, tanneries, magnetic tapes, paints, cement, copper industry, rubber industry, wood preservatives, cooling systems and as an additive to water to prevent corrosion [73, 129]. The Cr content in the sediment layer represents a permanent environmental risk because Cr is soluble, suspendable or transportable in water [129].

Pb concentrations in surface sediments ranged from 13.8 to 93.2 mg/kg, exceeding the shale value (20 mg/kg), indicating lead enrichment at the stations in the study area (Fig. 4). Pb is one of the toxic elements found naturally in the environment. In aquatic ecosystems, sources include the metal industry, mining, the paint industry, atmospheric deposition, and domestic and medical wastewater [43]. Pb entering the aquatic environment is generally immobile and tends to accumulate in the sediment layer near the point of entry [117] Sekhar et al.,[139].

In the study, Ni values ranged from 42.5 to 248.4 mg/kg, with an average of 136 ± 64.4 mg/kg. Although Ni values are below the threshold value (80 mg/kg- [89] at some stations, the average value is above the threshold value (Fig. 4). This situation indicates that some of the stations have become enriched in Ni. Ni is an element which is potentially dangerous and toxic. Entries into the environment occur through the metallurgical industry, fossil fuels, wastewater and geological decomposition products (Kahkönen and Kairesalo [82]; [50, 118].

The Mn value in metals ranged from 225.2 to 1167 mg/kg, and Mn values were found to be above the shale value (850 mg/kg) except for a few stations. This indicates that there is some enrichment of the Mn. Mn is one of the main elements sensitive to redox and Mn values higher than the shale average indicate the presence of both suboxic and anoxic conditions in the marine environment. Mn is an element that occurs naturally in the environment and is found in the earth’s crust at a concentration of approximately 0.1% [75]. Entry into aquatic environments occurs through mining, exhaust, agricultural pesticides, the steel industry, the glass industry, chemicals and batteries [178]. Mn entering surface waters precipitates in particulate form or oxidises and is adsorbed onto particles for vertical migration towards bottom sediments Williamson and Wilcock [172].

Fe ratios in surface sediments were found to be in the range of 1.8–10.6%, with an average of 6.2 ± 2.1. Since most stations and, in general, average Fe values are higher than shale values, this situation indicates anthropogenic effects in sediments (Fig. 4). Fe ranks fourth among metals in the Earth’s crust. It’s entries into the ecosystem occur anthropogenically through the iron and steel industry, wastewater, mining, fertilisers and pesticides [126]. In anaerobic conditions resulting from the remineralisation of organic matter in the sediment layer, iron ions can pass through pore water and their concentrations increase [29, 137]. Additionally, when sediment comes into contact with aerobic layers at or near the water interface, Fe ions are reduced and oxidised [59].

Within the scope of the study, Al percentages in surface sediments varied between 1.7 and 14.7%, with an average of 7.2 ± 3.9. The Al percentages remained below the shale percentage (9.2%) with the exception of a few stations. Al is the third most abundant metal in the Earth’s crust, accounting for 8.8% of its total composition. Al enters the environment naturally through the weathering of rocks and minerals. Anthropogenic sources include wastewater, air emissions and industrial processes, which are dispersed into the environment. Due to its prominence as a fundamental component of the earth’s crust, its entry into the environment through natural decomposition processes far exceeds its contribution to soil through air, water and human activities [94, 124].

Among the 10 metals determined in sediment samples taken from 15 different stations, the lowest value was 8.6 mg/kg and was detected at the Cr Fıstıklı Offshore (G4) station. The element with the highest concentration was Al, found at station 10 with 15.3%. All stations evaluated, the average values of the elements were ranked as Al > Fe > Mn > Zn > Cr > V > Ni > Cu > Pb > Co. The concentrations of Fe and Al are significantly higher than other elements, indicating that anthropogenic inputs are dominant in the environment.

Based on the shale averages provided in the Sea of Marmara Geochemistry Atlas published under the TÜBİTAK (Scientific and Technological Research Council of Turkey) Project previously, a significant enrichment of As, Pb, Sb, Mo, Ni, Cr, Zn and W metals has been reported in the Gemlik Bay [30].

The G10 station where sediment samples were collected is located near the point where the Karsak River flows into the river. Furthermore, the Gemlik Bay is affected by waste from the textile, metal, refinery, food (olive and olive oil), soap, and cement industries Akal-Solmaz et al., [4]. The major part of these wastes are transported to the Gemlik Bay via the Karsak River together with domestic wastes [30]. In recent years, the bay has been facing organic pollution due to domestic waste from the Karsak Çayı road and surrounding settlements, as well as waste from industries such as textiles, food and petroleum refineries. G11 is located in the area of Gemlik Port, while G13 is close to the outlet of the Kurşunlu Region deep sea discharge and is also a station with relatively dense settlement. All metals investigated in the study were found to be particularly high at these stations, which explains this situation.

The presence of Al at the highest levels in all stations, followed by Fe, suggests that this is the result of anthropogenic inputs. It was observed that Fe is the most concentrated trace element in all tissue samples and the highest values indicate that the environment is stressed.

[9] investigated the levels of aluminium (Al), iron (Fe), manganese (Mn), copper (Cu), lead (Pb), zinc (Zn), nickel (Ni), chromium (Cr), cobalt (Co) and mercury (Hg) in sediments of the Sea of Marmara. In the southern sediments, the values for Al and Fe were found to be 7.1% and 4%, respectively, while Mn, Cr, Ni, Co, Cu, Pb, Zn, and Hg were determined to be 504, 125, 67, 20, 31, 35, 131, and 0.5 mg/kg, respectively. Higher values in the south, particularly in the Gemlik and Erdek Bays, have been reported to originate from rock formations and mineralised areas. Additionally, high levels of Pb, Zn, and Cu in these areas have been determined to be related to anthropogenic pollution.

There have been numerous studies on metal accumulation in sediments in Turkish seas as well as in different regions and seas around the world. Instead of providing all the work that has been done, a few representative examples and a representative table comparing the data from these examples have been provided (Table 6). This table also includes two different studies conducted in the Gemlik Bay, which makes the comparison meaningful. The findings of our study across all metals are seen to be higher than the findings of other studies. Obviously, spatial differences play a role in these results, but the oceanographic conditions and variability of pollutant sources in the regions are also significant factors. Similarly, when the data from two separate studies published in 2008 and 2022 in the Gemlik Bay are taken into consideration, it is revealed that the data from this study is relatively low.

**Table 6:** Concentrations of metals in marine sediments from different regions of the world (mg/kg- dw)

Regions	Metals							References
	Cu	Cr	Ni	Mn	Pb	Zn	Fe	
San Fransisco Estuary, Brazil	11	53	19	17.9	12.9	131	26,000	[74]
Caspian Sea, Russia	8.3	-	14	-	4.19	17.1	5.5	[42]
Shelf sediments of Sea of Marmara, Türkiye*	28	130	56	300	29	84	2.9	[9]
Gemlik Bay*	38	145	128	727	39	122	4.4	[30]
Gemlik Bay*	58	181	165	1560	67	185	6.3	[164]
Bandırma Bay	8.9	61.2	-	-	27.8	38.7	-	[104]
Ambarlı Port	54	98	-	-	29	145	-	[133]
Black Sea, Romania	26.7	24.5	26.3	-	11.6	-	-	[79]
The Sea of Marmara	39.3	114.6	-	-	32.9	85.5	-	[111]
Black Sea Coast, Türkiye	576.3	-	-	-	97.3	357	-	[16]
Bohai Sea, China	39.8	175	-	-	18.6	117	-	[46]
Red Sea Coast, Saudi Arabia	18.7	-	13.7	-	3.56	16.8	1.4	[81]
Red Sea, Egyptian	9.4	-	17.5	198.8	11.4	44.2	8.45	[107]
Southeast coast, India	25.7	60	57.9	548.1	66.9	1.1	-	Satheeswaren et al., [135]
Black Sea, Bulgaria	19	-	18	-	19,75	-	-	Doncheva et al.[49]
Middle and Eastern Black Sea Coast, Türkiye	104.1	87.3	34.4	565.4	32.3	109.9	46	Apaydın et al., [11]
Eastern Sea area of Shendong Peninsula, China	19.5	22.6	-	-	15.5	26.1	-	[95]
Persian Gulf, Iran	22.5	31.4	95.8	511	5.77	39.4	22,000	[127]
Gemlik Bay	40	139.7	-	-	37.3	95.2	-	Arslan- Kaya et al., [12]
Mid-Black Sea, Türkiye	11.8	536.1	257.8	443.1	12.6	22.6	14.45	[112]
Gemlik Bay*	121.5	578.3	248.4	1167	93.2	358.8	10.56	This study

*: unit of Fe is given as %.

#### Sediment Quality Assessment

The metal accumulation status in surface sediments collected from different regions of Gemlik Bay was determined by measuring the concentrations of Cu, Zn, V, Co, Cr, Pb, Ni, Mn, Fe, and Al in the samples. The results were compared with the reference values published by Turekian and Wedepohl in 156 and Krauskoph in 89. Furthermore, this study examined metal concentrations in sediment samples using indices such as Igeo, EF, Cf, ER, PERI and PLI were used in indices and calculations, and findings were evaluated in terms of sediment quality. Total contamination factor (TCf) and modified contamination degree (*m*Cd) values were also shown in the Table 7.

**Table 7:** Cf, Er, TCf, *m*C*d*, PLI, PERI values of sediments

Stations	Contamination factor (Cf)										TCf	mCd	Ecological Risk (Er)					PLI	Metal	PERI
	Cu	Zn	V	Co	Cr	Pb	Ni	Mn	Fe	Al			Zn	Co	Cr	Pb	Ni			
G1	2.04	3.99	1.91	2.15	3.63	4.06	3.10	1.37	1.78	1.34	25.37	2.54	3.99	10.74	7.25	20.30	15.52	2.34	Zn	39.28
G2	1.70	3.23	1,47	1.70	3.00	3.19	2.50	0.97	1.37	0.29	19.42	1.94	3.23	8.50	6.01	15.96	12.48	1.62		
G3	1.73	2.46	1.55	1.50	2.06	1.67	1.54	0.87	1.47	0.70	15,54	1.55	2.46	7.48	4.11	8.33	7.71	1.34		
G4	0.40	0.86	0.38	0.43	0.83	0.69	0.66	0.26	0.39	0.31	5.22	0.52	0.86	2.14	1.66	3.46	3.32	0,48	Co	119.35
G5	1.30	2.46	1.28	1.74	2.87	2.98	2.62	0.92	1,42	0.72	18.31	1,83	2.46	8.68	5.75	14,92	13.10	1.65		
G6	0.72	1.62	0.63	0.86	1.30	2.39	0.94	0.77	0,64	0.45	10.31	1,03	1.62	4.31	2.60	11.97	4.68	0,91		
G7	1,35	3.15	1.48	1.43	1.41	4.66	0.53	1.34	1,53	1.10	17.97	1,80	3.15	7.13	2.82	23,30	2.65	1.53	Cr	74.26
G8	1.12	1.82	1.82	1.91	1.38	1.20	0.58	1.35	1,78	0.54	13.49	1,35	1.82	9.56	2.76	5.98	2.88	1.24		
G9	2.43	3.32	2.19	2.15	2.73	2.04	1.86	1.11	2.25	1.60	21.69	2.17	3.32	10.75	5.47	10.20	9.30	2.09		
G10	2.50	4.08	2.93	2.70	2.90	3.16	2.11	1.16	2.64	1.67	25.84	2.58	4.08	13.48	5.81	15.79	10.54	1.78	Pb	180.45
G11	1.38	3.37	1.37	1.56	2.46	2.68	2.06	1.07	1.46	0,74	18.14	1,81	3.37	7.78	4.91	13.38	10.28	1.66		
G12	0.98	1.64	1.09	1.34	1.88	0.95	1.72	0.77	1.13	0.85	12.35	1.24	1.64	6.71	3.77	4.77	8.61	1.18		
G13	0.68	1.72	1.36	1.36	1.45	1.50	1.01	1.07	1,32	0.69	12,15	1.21	1.72	6.79	2.89	7.48	5.07	1,16	Ni	128.27
G14	1.43	2.97	1.07	1.40	5,78	2.34	1.61	0.79	0.97	0.19	18,54	1.85	2.97	6.98	11.57	11.70	8.04	1,35		
G15	1.41	2.59	1.14	1.66	3,44	2.58	2.82	0.75	1.29	0.64	18,33	1.83	2.59	8.31	6.89	12.91	14.10	1.60		
Min	0.40	0.86	0.38	0.43	0.83	0.69	0.53	0.26	0.39	0.19	5.22	0.52	0.86	2.14	1.66	3.46	2.65	0.48		74.26
Max	2.50	4.08	2.19	2.15	5.78	4.66	3.10	1.37	2.64	1.67	25,84	2.58	4.08	10.75	11.57	23.30	15.52	2.34		180.45

Sediment quality guidelines (SQG) indicate that sediments at all stations are highly contaminated, particularly with Cr and Ni metals (Table 5). Moreover, with the exception of a few stations, the other stations are moderately to highly contaminated with Cu, Zn and Pb metals. According to the Canadian sediment quality guidelines (CCME), considering the specified limit values for Cu, Zn, Cr and Pb, the average values found for these metals in the sediment environment within the scope of the study are considerably high. When PEL values are evaluated overall, the highest values for Zn, Cr and Ni metals are recorded above the PEL values given in the table. This situation indicates that these metals are highly likely to have an adverse biological effect on benthic organisms in the region in question and that the sediment poses an ecotoxicological risk.

Sediment quality assessment includes single indices such as Igeo, EF and Cf, and multiple complex indices such as mCd and PLI to interpret the extent of metal contamination in sediments based on lithogenic and anthropogenic factors. [72] state that EF findings of 0.05 EF < 1.5 EF mean enrichment originating from lithogenic sources, natural processes or crustal materials, while EF > 1.5 means enrichment originating from anthropogenic factors.

Table 8 summarizes the EF values for metal contaminants in sediment samples. The EF values of surface sediments collected from the study area were 0.9–7.7 (mean 2.3–Cu), 1.9–16 (mean 4.6–Zn), 1.2–5.8 (mean 2.3–V), 1.3–7.5 (mean 2.7–Co), 0.1–31.1 (mean 5- Cr), 1.1–12.6 (mean 4.4- Pb), 0.5–8.7 (mean 3- Ni), 0.7–4.2 (mean 1.6- Mn) and 1.3–5.2 (mean 2.2- Fe). Cr demonstrated the greatest EF value among the ten metals examined and was categorized as seriously enriched, averaging an EF of 5. Cu, V, Co, Mn and Fe show a slight enrichment (average values are 2.3, 2.3, 2.7, 1.6 and 2.2, respectively), while Zn and Pb are classified as moderately enriched with values of 4.6 and 4.4, respectively. Even though enrichment factor values are evaluated based on averages, values below 1 at stations indicate natural accumulation.

**Table 8:** Igeo, EF, Cf, CaCO_3_, TOC values of sediments

Stations	Geoaccumulation index (Igeo)										Enrichment factor (EF)									CaCO3 %	TOC %
	Cu	Zn	V	Co	Cr	Pb	Ni	Mn	Fe	Al	Cu	Zn	V	Co	Cr	Pb	Ni	Mn	Fe		
G1	0.59	1.33	0.35	0.59	1.43	1.44	1.28	−0.13	0.24	0.04	1.5	3.0	1.4	1.6	2.7	3.0	2.3	1.0	1.3	15.10	4.74
G2	0.33	1.03	−0.02	0.25	1.15	1.09	0.97	−0.63	−0.14	−2.19	5.9	11.3	5.2	5.9	10.5	11.2	8.7	3.4	4.8	15.56	5.42
G3	0.36	0.64	0.05	0.07	0.61	0.15	0.27	−0.79	−0.04	−0,89	2.5	3.5	2.2	2.1	2.9	2.4	2.2	1.2	2.1	8.24	6.32
G4	−1.76	−0.87	−1.99	−1.73	−0.70	−1.12	−0.94	−2.50	−1.95	−2.07	1.3	2.8	1.2	1.4	2.7	2.2	2.1	0,9	1.3	15.10	6.12
G5	−0.05	0.64	−0.23	0.28	1.09	0.99	1.04	−0.71	−0.09	−0.85	1.8	3.4	1.8	2.4	4.0	4.1	3.6	1.3	2,0	18.31	4.21
G6	−0.90	0.03	−1.25	−0.73	−0.06	0.67	−0.45	−0.97	−1.23	−1.55	1.6	3.6	1.4	1.9	2.9	5.4	2.1	1.7	1,4	12.82	2.21
G7	0.00	0.99	−0.02	0.00	0.06	1.64	−1.26	−0.17	0.02	−0.25	1.2	2,9	1.3	1.3	1.3	4.2	0.5	1.2	1,4	4.73	2.82
G8	−0.26	0.20	0.28	0.42	0.03	−0.33	−1.15	−0.15	0.24	−1.27	2.1	3.4	3.4	3.5	2.6	2.2	1.1	2.5	3,3	3.20	3.47
G9	0.85	1.07	0.55	0.59	1.02	0.44	0.54	−0.43	0.58	0.29	1.5	2.1	1.4	1.3	1.7	1.3	1.2	0.7	1.4	6.41	4.47
G10	0.89	1.36	0.96	0.92	1.10	1.07	0.73	−0.37	0.81	0.35	1.5	2.4	1.8	1.6	1.7	1.9	1.3	0.7	1.6	14.65	5.11
G11	0.03	1.09	−0.13	0.13	0.86	0.83	0,69	−0.48	−0.04	−5.15	1.9	4.6	1.9	2.1	3.3	3.6	2.8	1.5	2.0	13.14	3.18
G12	−0.46	0.05	−0.47	−0.09	0.48	−0.65	0.43	−0.97	−0.42	−4.94	1.2	1.9	1.3	1.6	2.2	1.1	2.0	0.9	1.3	10.07	3.07
G13	−0.99	0.12	−0.15	−0.07	0.10	0,00	−0.33	−0.48	−0.20	−5.24	1.0	2.5	2.0	2.0	2.1	2.2	1.5	1.6	1.9	6.41	2.21
G14	0.08	0.91	−0.49	−0.03	2.10	0,64	0.33	−0.93	−0.63	−7.13	7.7	16.0	5.8	7.5	31.1	12.6	8.6	4.2	5.2	15.56	3.81
G15	0.06	0.71	−0.40	0.22	1.35	0.78	1.15	−0.99	−0.23	−5.35	2.2	4.1	1.8	2.6	5.4	4.0	4.4	1.2	2.0	10.53	3.85
Min	−1.76	−0.87	−1.99	−1.73	−0.70	−1.12	−1.26	−2.50	−1.95	−7.13	1.0	1.9	1.2	1.3	1.3	1.1	0.5	0.7	1.3	3.20	2.21
Max	0.89	1.36	0.96	0.92	2.10	1.64	1.28	−0.13	0.81	0.35	7.7	16.0	5.8	7.5	31.1	12.6	8.7	4.2	5.2	18.31	6.32

Based on the geochemical index calculations (Table 8), most stations and elements are classified as ‘unpolluted’ and ‘slightly polluted,’ while some stations are classified as ‘moderately polluted’ and ‘polluted’ in terms of Zn, Cu, and Pb.

Metals like Cu, Co, Zn, Pb, Cr and Ni and accumulate to a large extent on the main minerals of aluminosilicate and on the main elements determined by the hydrodynamic conditions at the working sites. These elements are primarily derived from the oxidation-reduction process of source rocks or terrigenous materials that have been weathered into metal oxides, hydroxides, oxyhydroxides and carbonates during the transport of sediment loads to coastal areas [19, 66, 158].

Table 7 displays the Cf values for metals in sediments. In this study, the highest Cf values were determined as Cr > Pb > Zn > Ni > Fe > Cu > V > Co > Al > Mn. However, since the Cf values for all metals and stations remained below 1, all stations were classified as low contamination.

The results of other calculated values, namely potential ecological risk (ER) and index (PERI), are presented in Table 7. At G7 (Kapaklı) station, since the Er values for all metals except Pb remained below 20, the ecological risk factor for each metal is classified as low potential. In contrast, all calculated risk indices for metals are classified as low risk, as they are all below 30.

The TCf, *m*Cd and PLI values calculated for metals are shown in Table 7. The G4 (Fıstıklı offshore) station, found to be TCf < 6, is low risk, while the G1 (Kurşunlu Offshore) and G10 (Karsak River Mouth) stations, found to be TCf > 25, represent very high pollution, and the values at other stations, ranging from 12 < TCf < 24, indicate quite high pollution. With the exception of stations G1, G9 and G10, which were found to have *m*C*d* values of < 2 and were moderately contaminated, the *m*C*d* values of the other stations were found to be below 2, indicating low and very low levels of contamination. However, PLI values were found to be in the range of 0.48–2.34 across all stations. No pollution was observed at stations G4 and G6 (PLI < 1), while high pollution (2 < PLI < 3) was observed at stations G1 and G9. At other stations, PLI values were between 1 and 2, indicating moderate pollution.

CaCO_3_ values varied between 3.20% and 18.31% across stations (Table 8). According to the geochemistry atlas, Total Carbonate (Tkarb) is low in the Gemlik bays, reported to be approximately 10% CaCO_3_, and it has been stated that the most important source of high Tkarb values is biogenic carbonate shells and shell fragments (Çağatay et al., 2025). The high CaCO_3_ values in this study suggest a correlation with this condition.

The geochemistry atlas reports that the average C*org* content range in the Sea of Marmara is 0.04%–6.21%, and that C*org* values in the gulfs are above this average (Çağatay et al., 2025). Within the scope of this study, TOC values were found to be in the range of 2.21–6.32%, confirming this situation (Table 8).

### Biota-Sediment Accumulation Factor (BSAF)

Algae, with their significant metal bioaccumulation capacity, serve as valuable bioindicators in the evaluation of marine ecosystems. The ability of some algae to endure polluted environments is linked to genetic mutations they possess. In this study, BSAF values related to sediment were calculated in both mussels and algae. BSAF determines the efficiency of plants in accumulating elements present in the environment and evaluates the transfer and mobilization of these elements from environmental sources to plant tissues [3]. When the bioaccumulation factor (BSAF) exceeds 1, the element tends to accumulate in plant tissues [51, 122].

The number and locations of stations where sediments and biota were collected varied considerably, but biota-sediment accumulation factors (BSAFs) were calculated based on dry weights for several stations located close to each other. Accordingly, the stations that are close to each other, as shown in Fig. 1, are sediment (G) and biota (K) stations that are close to each other; G1- K1, G8- K2, G12- K4, G13- K5, G15- K7. Accordingly, the biota-sediment accumulation factors (BSAFs) calculated separately for mussels and macroalgae are presented in Table 9.

**Table 9:** Biota-sediment transfer factors (BSAFs) in organisms sampled at joint stations in Gemlik Bay

BSAFs for mussels								
Metals Stations	Cu	Zn	V	Co	Cr	Pb	Ni	Mn
K1 vs. G1	0.23	**4.57**	-	0.11	0.02	-	0.05	0.04
K2 vs. G8	**1.61**	**2.03**	-	-	-	-	-	0.01
K4 vs. G12	0.29	**4.98**	-	-	0.03	-	0.08	0.05
K5 vs. G13	0.59	**6.53**	-	0.14	0.07	-	0.15	0.05
K7 vs. G15	**1.77**	**3.22**	-	-	0.02	-	0.05	0.03
**BSAFs for seaweeds**								
**Metals** **Stations**	**Cu**	**Zn**	**V**	**Co**	**Cr**	**Pb**	**Ni**	**Mn**
K1 vs. G1	0.27	0.09	0.04	-	0.03	-	0.06	0.13
K2 vs. G8	0.26	0.12	-	-	-	-	-	0.02
K4 vs. G12	0.34	0.50	0.07	-	0.07	-	0.14	0.43
K5 vs. G13	**2.48**	**2.13**	**1.21**	**1**,**49**	**2.14**	**2.10**	**2.65**	0.98
K7 vs. G15	0.62	0.19	0.10	-	0.02	-	0.04	0.10

Values in bold indicate the transfer of pollutants from sediment to biota (BSAF > 1).

BSAF < 1 for *Ulva lactuca*, except for station K5, indicates that there is no bioaccumulation from sediment (Table 9). Similar conditions have been observed in studies conducted off the coast of Senegal [47] and Lebanon [62]. Some studies have identified statistically significant correlations between trace element concentrations in *U. lactuca* and those in water and sediments (Akcali and Kucuksezgin, [5]; [26, 34]. However, other investigations have reported no significant associations between trace element levels in algae and environmental matrices [83, 100].

As can be seen in Table 9 and as indicated in bold, In mussels, Zn shows BSAF > 1 at all sampling stations, as well as Cu at stations K2-G8 and K7-G15. Cu and Zn elements can be found together. Furthermore, Kurşunlu station is one of the points exposed to maritime traffic and is located close to a deep sea discharge point. The high concentration of Zn in mussels compared to other metal values indicates that this affects metal interactions in sediments and macroalgae. The proximity of these stations to the source of pollution explains the high values found in all metal values in algae, except for Mn. Since Mn precipitates in water as oxidised forms, other metals are more abundant in water. Low Mn values have also been linked to algae and mussels feeding on water in aquatic environments.

Metals found in marine organisms are derived not solely from sediments but also from different sources. Bioaccumulation is influenced by several factors such as bioavailability, multiple exposure routes, and metabolic differences among species (Jakimska et al., 2011). Due to the heterogeneous uptake of trace elements, it is crucial to incorporate multiple taxa and distinct tissues within the same organism when designing monitoring and bioaccumulation studies [183].

### Potential Sources of Metals

The normality and equality of variance of the data were tested using the Kolmogorov–Smirnov and Shapiro-Wilk test (*p* < 0.05). The relationship between metal concentrations obtained from mussel and algae samples and sediment content was tested by principal component analysis (PCA). The Kaiser-Meyer Olkin (KMO) test result for PCA sampling adequacy was found to be 0.859, indicating “excellent” adequacy (sig. of Bartlett’s Test of Sphericity < 0.001). According to the eigenvalues ​​obtained as a result of the analysis, it is seen that the first two components (PC1 and PC2) explain 87.085% (78.226% + 8.859%) of the total variance. As demonstrated in Table 10 (factor loadings), the variables V, Co, Cr, Pb, Ni, Mn, Al, Fe, CaCO_3_%, TOC% and depth are considered to be high, and Cu is identified as moderately important for PC1. Additionally, Zn is categorised as high and Cu as moderately important for PC2. In relation to PC2, other variables were represented by lower values. As indicated by the Spearman correlation matrix (Table 11), all variables with the exception of Zn demonstrate a high correlation with each other (**: *p* < 0.0, *: *p* < 0.05).

**Table 10:** PCA’s factor loadings

Variables	PC1	PC2
**Cu**	0.640	0.386
**Zn**	−0.022	0.973
**V**	0.960	−0.102
**Co**	0.932	0.206
**Cr**	0.945	0.143
**Pb**	0.959	0.120
**Ni**	0.893	0.167
**Mn**	0.935	−0.052
**Al%**	0.924	0.060
**Fe%**	0.951	0.076
**CaCO**_**3**_ **%**	0.933	0.004
**TOC%**	0.945	0.009
**Depth**	0.946	−0.011

**Table 11:** Spearman rank correlation matrix for between dissolved metal concentrations with CaCO_3_%, TOC% and depth (**: *p* < 0.0, *: *p* < 0.05)

Variables		Cu	Zn	V	Co	Cr	Pb	Ni	Mn	Al%	Fe%	CaCO_3_%	TOC%	Depth
**Cu**		1	0.320	0.650**	0.653**	0.586**	0.626**	0.573**	0.510**	0.558**	0.600**	0.471*	0.548**	0.501**
**Zn**		0.320	1	−0.047	0.183	0.063	0.119	0.048	−0.009	0.015	0.040	−0.050	−0.037	−0.082
**V**		0.650**	−0.047	1	0.921**	0.831**	0.857**	0.828**	0.941**	0.825**	0.879**	0.651**	0.761**	0.721**
**Co**		0.653**	0.183	0.921**	1	0.882**	0.878**	0.887**	0.875**	0.799**	0.853**	0.709**	0.771**	0.728**
**Cr**		0.586**	0.063	0.831**	0.882**	1	0.866**	0.961**	0.821**	0.706**	0.721**	0.781**	0.747**	0.738**
**Pb**		0.626**	0.119	0.957**	0.878**	0.866**	1	0.862**	0.855**	0.789**	0.801**	0.785**	0.741**	0.766**
**Ni**		0.573**	0.048	0.828**	0.887**	0.961**	0.862**	1	0.797**	0.728**	0.712**	0.776**	0.744**	0.714**
**Mn**		0.510**	−0.009	0.041**	0.875**	0.821**	0.855**	0.797**	1	0.818**	0.872**	0.657**	0.699**	0.697**
**Al%**		0.558**	0.015	0.825**	0.799**	0.706**	0.789**	0.728**	0.818**	1	0.951**	0.783**	0.832**	0.782**
**Fe%**		0.600**	0.040	0.879**	0.853**	0.721**	0.801**	0.712**	0.872**	0.951**	1	0.773**	0.869**	0.832**
**CaCO**_**3**_ **%**		0.471*	−0.050	0.651**	0.709**	0.781**	0.785**	0.776**	0.657**	0.783**	0.773**	1	0.901**	0.914**
**TOC%**		0.548**	−0.037	0.761**	0.771**	0.747**	0.741**	0.744**	0.699**	0.832**	0.869**	0.901**	1	0.963**
**Depth**		0.501**	−0.082	0.721**	0.728**	0.738**	0.766**	0.714**	0.697**	0.782**	0.832**	0.914**	0.963**	1

**Fig. 5:**
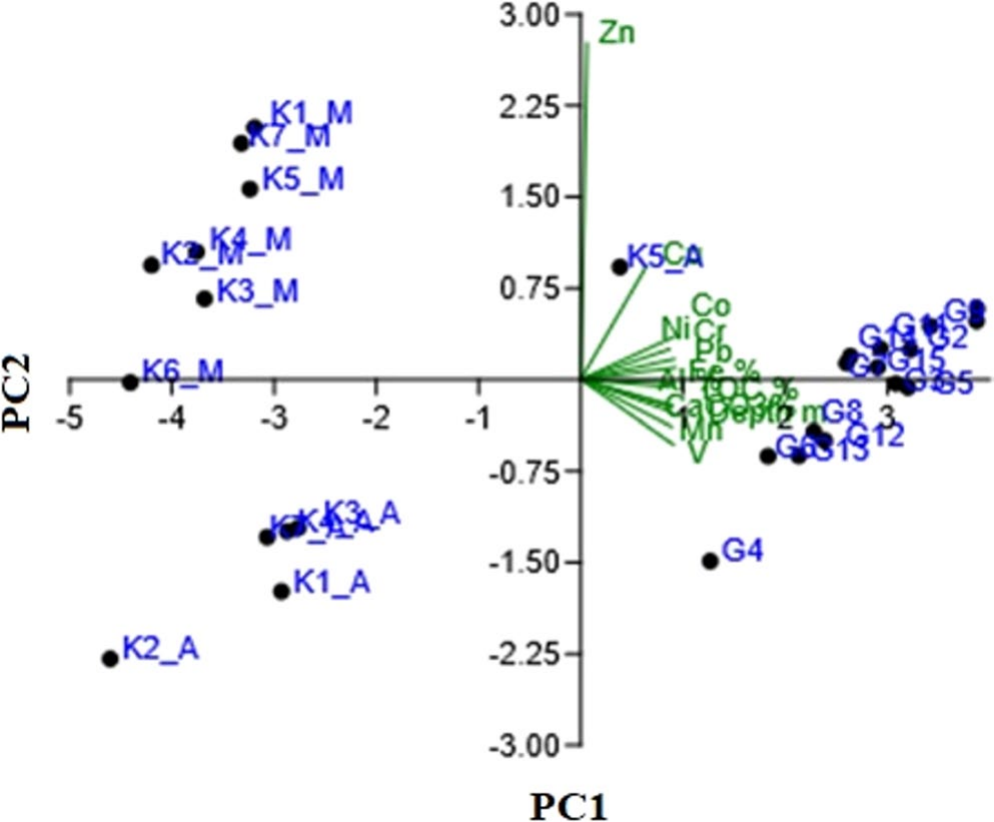
PCA analysis graph variables and stations (variables highlighted in green, stations highlighted in blue)

The elevated correlation between Cu, Cr, Pb and Ni signifies the presence of pollutants originating from industrial and urban discharges, as well as port activities. These metals are also related to the heavy industry (paint, petrochemical, etc.) located around the Gemlik Bay. On the other hand, these metals whose existence has been investigated are similar to each other with similar geochemical-geological processes. These particles bind to organic matter, and as a result of the sedimentation process, they settle out of the water column and begin to accumulate in the sediment. The PCA results also explain the natural, geological and anthropogenic effects of these metals, which are especially associated with PC1, and sedimentation processes together with TOC and depth. Among these metals, only Zn, which is associated with PC2, has been shown to be indicative of anthropogenic and industrial pollutants (Table 10; Fig. 5).

Additionally, as seen from the PCA biplot, the stations where sediment samples were taken reflect high contamination levels in relation to PC1. It has been observed that the samples obtained from mussel contents are heavily influenced by Zn, while the values obtained from algae samples are not affected by both components. The Zn effect was also obtained from mussel samples obtained from coastal areas, thus reflecting the effect of only anthropogenic and local urban discharges at these coastal stations. The stations where sediment samples highly correlated with PC1 were obtained are offshore and their depths vary between 34 and 108 m. In this case, it means that in Gemlik Bay, where metal pollutants are high, the retention in the sediment increases with depth.

## Conclusion

This study provides detailed information and risk assessments regarding the distribution of metal concentrations (Fe, Al, Ni, Cu, Co, Zn, Pb and Cr) in sediments, mussels (*M. galloprovincialis*), and macroalgae (*Ulva lactuca*,* Ulva intestinalis*) in Gemlik Bay. Metal concentrations in all sample types were generally higher than those reported in other regions worldwide. Moreover, the detected metals in mussel and macroalgae samples were evaluated considering four separate risk groups for possible consumption, and it was concluded that children are at a relatively higher risk. It would therefore be appropriate to emphasise that greater care should be taken with regard to children’s consumption.

Integrated Pollution Monitoring Studies have been conducted in our country’s seas for many years by the T.C. Ministry of Environment, Urbanisation and Climate Change. In this study, a detailed and specific risk assessment was carried out for different age groups using findings obtained from Gemlik Bay, presenting a rich data set for the study. Furthermore, the fact that the findings are significantly higher than in previous studies highlights that this region has been exposed to serious pollution over time. Thus, this study will also confirm the hypothesis that pollution must be brought under control in Gemlik Bay, which has been exposed to human-induced pollution for many years. 

## Supplementary Information

Below is the link to the electronic supplementary material.

Supplementary Material 1

## Data Availability

No datasets were generated or analysed during the current study.
