# Supplementary material for: Spatial Variability of Metal Concentrations in Seaweeds, Mussels and Surface Sediments in Gemlik Bay: an Extensive Assessment of Contamination Sources and Associated Health Risks
Source: Biol Trace Elem Res. 2026 Feb 3;204(6):4323–47. doi: 10.1007/s12011-025-04967-6 (PMC13157430; doi:10.1007/s12011-025-04967-6)
Supplement: Supplementary file 1 — Supplementary Material 1 [file 12011_2025_4967_MOESM1_ESM.docx]

Table S1: Mussel and macroalgae stations and coordinates in Gemlik Bay

| **Station no** | **Station name** | **Coordinates** |
| --- | --- | --- |
| K1 | Armutlu | 40° 23' 42.6" 29° 01' 13.8" |
| K2 | Narlı | 40° 28' 45.9" 29° 01' 45.2" |
| K3 | Kapaklı | 40° 27' 44" 29° 06' 23.8" |
| K4 | Gemlik Port | 40° 27' 44" 29° 06' 23.8" |
| K5 | Kurşunlu Pier | 40° 22' 00.7" 29° 03' 42.2" |
| K6 | Mudanya Port | 40° 22' 59.2" 28° 52' 20.9" |
| K7 | Kumyaka | 40° 23' 01.6" 28° 49' 45.7" |

Table S2: Sediments stations and coordinates in Gemlik Bay

| **Station no** | **Station name** | **Coordinates** | **Depth (m)** |
| --- | --- | --- | --- |
| G1 | Kurşunlu Offshore | 40° 23' 42.6"- 29° 01' 13.8" | 90 m |
| G2 | Kurşunlu West | 40° 22' 43.3"- 28° 58' 58.9" | 99.5 m |
| G3 | Burgaz Çukuru | 40° 23' 34.8"- 28° 57' 03.5" | 108 m |
| G4 | Fıstıklı Offshore | 40° 26' 52.6"- 28° 52' 24" | 90 m |
| G5 | Fıstıklı | 40° 28' 54.2"- 28° 52' 24" | 60 m |
| G6 | Armutlu | 40° 29' 41.2"- 28° 48' 26.6" | 36 m |
| G7 | Kapaklı | 40° 27' 35.4"- 28° 57' 48.2" | 36 m |
| G8 | Narlı | 40° 28' 41.1"- 29° 02' 00.4" | 36 m |
| G9 | Küçükkumla | 40° 28' 31.1"- 29° 03' 40.4" | 36 m |
| G10 | Karsak River Mouth | 40° 25' 49.4"- 29° 08' 37.6" | 36 m |
| G11 | Gemlik -Port | 40° 25' 08.5"- 29° 06' 14.6" | 34 m |
| G12 | RODAPORT- Port | 40° 25' 05.0"- 29° 04' 30.6" | 34 m |
| G13 | Kurşunlu | 40° 21' 48.3"- 29° 01' 17.3" | 35 m |
| G14 | Mudanya | 40° 22' 20.1"- 28° 54' 24.4" | 45 m |
| G15 | Tirilye-Kumyaka | 40° 23' 55.6"- 28° 48' 30.2" | 36 m |

Table S3: CDI values for seaweeds

| Metals | | Cu | | | | Zn | | | | Co | | | |
| --- | --- | --- | --- | --- | --- | --- | --- | --- | --- | --- | --- | --- | --- |
| Groups | | C | A | WA | MA | C | A | WA | MA | C | A | WA | MA |
| No | Stations |  |  |  |  |  |  |  |  |  |  |  |  |
| 1 | Armutlu | 1.37E+00 | 3.93E-01 | 4.92E-02 | 7.00E-02 | 1.61E+00 | 4.64E-01 | 5.80E-02 | 8.25E-02 | - | - | - | - |
| 2 | Narlı | 7.27E-01 | 2.09E-01 | 2.62E-02 | 3.72E-02 | 9.73E-01 | 2.80E-01 | 3.50E-02 | 4.97E-02 | - | - | - | - |
| 3 | Küçükkumla | 8.91E-01 | 2.56E-01 | 3.21E-02 | 4.56E-02 | 2.97E+00 | 8.53E-01 | 1.07E-01 | 1.52E-01 | 1.86E-01 | 5.35E-02 | 6.69E-03 | 9.51E-03 |
| 4 | Gemlik Port | 8.22E-01 | 2.36E-01 | 2.96E-02 | 4.21E-02 | 3.61E+00 | 1.04E+00 | 1.30E-01 | 1.85E-01 | - | - | - | - |
| 5 | Kurşunlu Pier | 4.16E+00 | 1.20E+00 | 1.50E-01 | 2.13E-01 | 1.62E+01 | 4.67E+00 | 5.84E-01 | 8.30E-01 | 1.99E+00 | 5.71E-01 | 7.15E-02 | 1.02E-01 |
| 7 | Kumyaka | 2.14E+00 | 6.16E-01 | 7.72E-02 | 1.10E-01 | 2.15E+00 | 6.19E-01 | 7.75E-02 | 1.10E-01 | - | - | - | - |
|  | |  |  |  |  |  |  |  |  |  |  |  |  |
| Metals | | Cr | | | | Ni | | | | Mn | | | |
| Groups | | C | A | WA | MA | C | A | WA | MA | C | A | WA | MA |
| No | Stations |  |  |  |  |  |  |  |  |  |  |  |  |
| 1 | Armutlu | 4.99E-01 | 1.43E-01 | 1.79E-02 | 2.55E-02 | 7.62E-01 | 2.19E-01 | 2.74E-02 | 3.90E-02 | 7.41E+00 | 2.13E+00 | 2.66E-01 | 3.79E-01 |
| 2 | Narlı | - | - | - | - | - | - | - | - | 1.24E+00 | 3.56E-01 | 4.45E-02 | 6.33E-02 |
| 3 | Küçükkumla | 5.68E-01 | 1.63E-01 | 2.04E-02 | 2.90E-02 | 9.49E-01 | 2.73E-01 | 3.42E-02 | 4.86E-02 | 4.78E+00 | 1.37E+00 | 1.72E-01 | 2.44E-01 |
| 4 | Gemlik Port | 6.46E-01 | 1.86E-01 | 2.33E-02 | 3.30E-02 | 9.69E-01 | 2.79E-01 | 3.49E-02 | 4.96E-02 | 1.37E+01 | 3.94E+00 | 4.93E-01 | 7.01E-01 |
| 5 | Kurşunlu Pier | 1.53E+01 | 4.39E+00 | 5.49E-01 | 7.80E-01 | 1.06E+01 | 3.04E+00 | 3.80E-01 | 5.40E-01 | 4.38E+01 | 1.26E+01 | 1.58E+00 | 2.24E+00 |
| 7 | Kumyaka | 3.23E-01 | 9.29E-02 | 1.16E-02 | 1.65E-02 | 4.90E-01 | 1.41E-01 | 1.76E-02 | 2.50E-02 | 3.07E+00 | 8.84E-01 | 1.11E-01 | 1.57E-01 |

C: Child, A: Adolescent, WA: Woman adult, MA: Man adult, “-“: not calculated due to below detection limit of elements

Table S4: EWI values for seaweeds

| Metals | | Cu | | | | Zn | | | | Co | | | |
| --- | --- | --- | --- | --- | --- | --- | --- | --- | --- | --- | --- | --- | --- |
| Groups | | C | A | WA | MA | C | A | WA | MA | C | A | WA | MA |
| No | Stations |  |  |  |  |  |  |  |  |  |  |  |  |
| 1 | Armutlu | 9.58E+00 | 2.75E+00 | 3.45E-01 | 4.90E-01 | 1.13E+01 | 3.25E+00 | 4.06E-01 | 5.77E-01 | - | - | - | - |
| 2 | Narlı | 5.09E+00 | 1.46E+00 | 1.83E-01 | 2.60E-01 | 6.81E+00 | 1.96E+00 | 2.45E-01 | 3.48E-01 | - | - | - | - |
| 3 | Küçükkumla | 6.24E+00 | 1.79E+00 | 2.24E-01 | 3.19E-01 | 2.08E+01 | 5.97E+00 | 7.47E-01 | 1.06E+00 | 1.30E+00 | 3.74E-01 | 4.68E-02 | 6.66E-02 |
| 4 | Gemlik Port | 5.76E+00 | 1.65E+00 | 2.07E-01 | 2.94E-01 | 2.53E+01 | 7.27E+00 | 9.10E-01 | 1.29E+00 | - | - | - | - |
| 5 | Kurşunlu Pier | 2.91E+01 | 8.37E+00 | 1.05E+00 | 1.49E+00 | 1.14E+02 | 3.27E+01 | 4.09E+00 | 5.81E+00 | 1.39E+01 | 4.00E+00 | 5.00E-01 | 7.11E-01 |
| 7 | Kumyaka | 1.50E+01 | 4.32E+00 | 5.40E-01 | 7.68E-01 | 1.51E+01 | 4.34E+00 | 5.43E-01 | 7.71E-01 | - | - | - | - |
|  | |  |  |  |  |  |  |  |  |  |  |  |  |
| Metals | | Cr | | | | Ni | | | | Mn | | | |
| Groups | | C | A | WA | MA | C | A | WA | MA | C | A | WA | MA |
| No | Stations |  |  |  |  |  |  |  |  |  |  |  |  |
| 1 | Armutlu | 3.49E+00 | 1.00E+00 | 1.26E-01 | 1.78E-01 | 5.34E+00 | 1.53E+00 | 1.92E-01 | 2.73E-01 | 5.18E+01 | 1.49E+01 | 1.87E+00 | 2.65E+00 |
| 2 | Narlı | - | - | - | - | - | - | - | - | 8.66E+00 | 2.49E+00 | 3.12E-01 | 4.43E-01 |
| 3 | Küçükkumla | 3.97E+00 | 1.14E+00 | 1.43E-01 | 2.03E-01 | 6.65E+00 | 1.91E+00 | 2.39E-01 | 3.40E-01 | 3.34E+01 | 9.61E+00 | 1.20E+00 | 1.71E+00 |
| 4 | Gemlik Port | 4.52E+00 | 1.30E+00 | 1.63E-01 | 2.31E-01 | 6.78E+00 | 1.95E+00 | 2.44E-01 | 3.47E-01 | 9.59E+01 | 2.76E+01 | 3.45E+00 | 4.91E+00 |
| 5 | Kurşunlu Pier | 1.07E+02 | 3.07E+01 | 3.84E+00 | 5.46E+00 | 7.40E+01 | 2.13E+01 | 2.66E+00 | 3.78E+00 | 3.07E+02 | 8.82E+01 | 1.10E+01 | 1.57E+01 |
| 7 | Kumyaka | 2.26E+00 | 6.50E-01 | 8.14E-02 | 1.16E-01 | 3.43E+00 | 9.85E-01 | 1.23E-01 | 1.75E-01 | 2.15E+01 | 6.19E+00 | 7.74E-01 | 1.10E+00 |

C: Child, A: Adolescent, WA: Woman adult, MA: Man adult, “-“: not calculated due to below detection limit of elements

Table S5: THQ and HI values for seaweeds

| St. No | Risk group | **Target Hazard Quotient (THQ)** | | | | | | **HI** |
| --- | --- | --- | --- | --- | --- | --- | --- | --- |
|  |  | Cu | Zn | Co | Cr | Ni | Mn |  |
| 1 | Child | 2.74E+00 | 5.38E+00 | - | 1.66E+02 | 3.81E+01 | 5.29E+01 | 265.30 |
|  | Adolescent | 7.87E-01 | 1.55E+00 | - | 4.78E+01 | 1.10E+01 | 1.52E+01 | 76.27 |
|  | Woman | 9.85E-02 | 1.93E-01 | - | 5.98E+00 | 1.37E+00 | 1.90E+00 | 9.55 |
|  | Man | 1.40E-01 | 2.75E-01 | - | 8.50E+00 | 1.95E+00 | 2.71E+00 | 13.57 |
| 2 | Child | 1.45E+00 | 3.24E+00 | - | - | 0.00E+00 | 8.84E+00 | 13.54 |
|  | Adolescent | 4.18E-01 | 9.32E-01 | - | - | 0.00E+00 | 2.54E+00 | 3.89 |
|  | Woman | 5.23E-02 | 1.17E-01 | - | - | 0.00E+00 | 3.18E-01 | 0.49 |
|  | Man | 7.43E-02 | 1.66E-01 | - | - | 0.00E+00 | 4.52E-01 | 0.69 |
| 3 | Child | 1.78E+00 | 9.89E+00 | 6.20E+00 | 1.89E+02 | 4.75E+01 | 3.41E+01 | 288.71 |
|  | Adolescent | 5.12E-01 | 2.84E+00 | 1.78E+00 | 5.44E+01 | 1.36E+01 | 9.81E+00 | 83.00 |
|  | Woman | 6.41E-02 | 3.56E-01 | 2.23E-01 | 6.81E+00 | 1.71E+00 | 1.23E+00 | 10.39 |
|  | Man | 9.11E-02 | 5.06E-01 | 3.17E-01 | 9.68E+00 | 2.43E+00 | 1.74E+00 | 14.76 |
| 4 | Child | 1.64E+00 | 1.20E+01 | - | 2.15E+02 | 4.85E+01 | 9.79E+01 | 375.45 |
|  | Adolescent | 4.73E-01 | 3.46E+00 | - | 6.19E+01 | 1.39E+01 | 2.81E+01 | 107.93 |
|  | Woman | 5.92E-02 | 4.33E-01 | - | 7.75E+00 | 1.74E+00 | 3.52E+00 | 13.51 |
|  | Man | 8.41E-02 | 6.16E-01 | - | 3.88E-01 | 2.48E+00 | 5.01E+00 | 8.57 |
| 5 | Child | 8.32E+00 | 5.41E+01 | 6.62E+01 | 5.09E+03 | 5.28E+02 | 3.13E+02 | 6056.88 |
|  | Adolescent | 2.39E+00 | 1.56E+01 | 1.90E+01 | 1.46E+03 | 1.52E+02 | 9.00E+01 | 1741.21 |
|  | Woman | 2.99E-01 | 1.95E+00 | 2.38E+00 | 1.83E+02 | 1.90E+01 | 1.13E+01 | 217.94 |
|  | Man | 4.25E-01 | 2.77E+00 | 3.39E+00 | 2.60E+02 | 2.70E+01 | 1.60E+01 | 309.74 |
| 7 | Child | 4.29E+00 | 7.18E+00 | - | 1.08E+02 | 2.45E+01 | 2.20E+01 | 165.63 |
|  | Adolescent | 1.23E+00 | 2.06E+00 | - | 3.10E+01 | 7.04E+00 | 6.31E+00 | 47.61 |
|  | Woman | 1.54E-01 | 2.58E-01 | - | 3.88E+00 | 8.81E-01 | 7.90E-01 | 5.96 |
|  | Man | 2.19E-01 | 3.67E-01 | - | 5.51E+00 | 1.25E+00 | 1.12E+00 | 8.47 |

Table S6: CDI values for mussels

| Metals | | Cu | | | | Zn | | | | Co | | | |
| --- | --- | --- | --- | --- | --- | --- | --- | --- | --- | --- | --- | --- | --- |
| Groups | | C | A | WA | MA | C | A | WA | MA | C | A | WA | MA |
| No | Stations |  |  |  |  |  |  |  |  |  |  |  |  |
| 1 | Armutlu | 1.54E-01 | 4.41E-02 | 5.53E-03 | 7.85E-03 | 1.06E+01 | 3.04E+00 | 3.81E-01 | 5.42E-01 | 3.22E-02 | 9.25E-03 | 1.16E-03 | 1.65E-03 |
| 2 | Narlı | 5.83E-01 | 1.68E-01 | 2.10E-02 | 2.98E-02 | 2.15E+00 | 6.18E-01 | 7.73E-02 | 1.10E-01 | - | - | - | - |
| 3 | Küçükkumla | 6.89E-02 | 1.98E-02 | 2.48E-03 | 3.53E-03 | 3.90E+00 | 1.12E+00 | 1.40E-01 | 1.99E-01 | - | - | - | - |
| 4 | Gemlik Port | 9.25E-02 | 2.66E-02 | 3.33E-03 | 4.73E-03 | 4.74E+00 | 1.36E+00 | 1.71E-01 | 2.43E-01 | - | - | - | - |
| 5 | Kurşunlu Pier | 1.30E-01 | 3.75E-02 | 4.69E-03 | 6.67E-03 | 6.53E+00 | 1.88E+00 | 2.35E-01 | 3.34E-01 | 2.45E-02 | 7.04E-03 | 8.82E-04 | 1.25E-03 |
| 6 | Mudanya Port | 6.17E-02 | 1.77E-02 | 2.22E-03 | 3.16E-03 | 1.72E+00 | 4.93E-01 | 6.18E-02 | 8.78E-02 | - | - | - | - |
| 7 | Kumyaka | 8.03E-01 | 2.31E-01 | 2.89E-02 | 4.11E-02 | 4.85E+00 | 1.39E+00 | 1.75E-01 | 2.48E-01 | - | - | - | - |
|  | |  |  |  |  |  |  |  |  |  |  |  |  |
| Metals | | Cr | | | | Ni | | | | Mn | | | |
| Groups | | C | A | WA | MA | C | A | WA | MA | C | A | WA | MA |
| No | Stations |  |  |  |  |  |  |  |  |  |  |  |  |
| 1 | Armutlu | 5.29E-02 | 1.52E-02 | 1.91E-03 | 2.71E-03 | 7.84E-02 | 2.25E-02 | 2.82E-03 | 4.01E-03 | 2.71E-01 | 7.79E-02 | 9.76E-03 | 1.39E-02 |
| 2 | Narlı | - | - | - | - | - | - | - | - | 7.81E-02 | 2.25E-02 | 2.81E-03 | 4.00E-03 |
| 3 | Küçükkumla | 4.31E-02 | 1.24E-02 | 1.55E-03 | 2.20E-03 | 4.96E-02 | 1.42E-02 | 1.78E-03 | 2.53E-03 | 2.48E-01 | 7.12E-02 | 8.91E-03 | 1.27E-02 |
| 4 | Gemlik Port | 4.11E-02 | 1.18E-02 | 1.48E-03 | 2.10E-03 | 6.90E-02 | 1.98E-02 | 2.48E-03 | 3.53E-03 | 2.08E-01 | 5.99E-02 | 7.50E-03 | 1.07E-02 |
| 5 | Kurşunlu Pier | 6.57E-02 | 1.89E-02 | 2.37E-03 | 3.36E-03 | 8.02E-02 | 2.31E-02 | 2.89E-03 | 4.10E-03 | 2.84E-01 | 8.17E-02 | 1.02E-02 | 1.45E-02 |
| 6 | Mudanya Port | - | - | - | - | 3.86E-02 | 1.11E-02 | 1.39E-03 | 1.97E-03 | 9.55E-02 | 2.74E-02 | 3.43E-03 | 4.88E-03 |
| 7 | Kumyaka | 3.68E-02 | 1.06E-02 | 1.32E-03 | 1.88E-03 | 6.59E-02 | 1.89E-02 | 2.37E-03 | 3.37E-03 | 1.12E-01 | 3.21E-02 | 4.01E-03 | 5.70E-03 |

C: Child, A: Adolescent, WA: Woman adult, MA: Man adult, “-“: not calculated due to below detection limit of elements

Table S7: EWI values for mussels

| Metals | | Cu | | | | Zn | | | | Co | | | |
| --- | --- | --- | --- | --- | --- | --- | --- | --- | --- | --- | --- | --- | --- |
| Groups | | C | A | WA | MA | C | A | WA | MA | C | A | WA | MA |
| No | Stations |  |  |  |  |  |  |  |  |  |  |  |  |
| 1 | Armutlu | 1.08E+00 | 3.09E-01 | 3.87E-02 | 5.50E-02 | 7.41E+01 | 2.13E+01 | 2.67E+00 | 3.79E+00 | 2.25E-01 | 6.48E-02 | 8.11E-03 | 1.15E-02 |
| 2 | Narlı | 4.08E+00 | 1.17E+00 | 1.47E-01 | 2.09E-01 | 1.50E+01 | 4.32E+00 | 5.41E-01 | 7.69E-01 | - | - | - | - |
| 3 | Küçükkumla | 4.83E-01 | 1.39E-01 | 1.74E-02 | 2.47E-02 | 2.73E+01 | 7.84E+00 | 9.82E-01 | 1.40E+00 | - | - | - | - |
| 4 | Gemlik Port | 6.48E-01 | 1.86E-01 | 2.33E-02 | 3.31E-02 | 3.32E+01 | 9.54E+00 | 1.19E+00 | 1.70E+00 | - | - | - | - |
| 5 | Kurşunlu Pier | 9.12E-01 | 2.62E-01 | 3.28E-02 | 4.67E-02 | 4.57E+01 | 1.31E+01 | 1.64E+00 | 2.34E+00 | 1.72E-01 | 4.93E-02 | 6.17E-03 | 8.77E-03 |
| 6 | Mudanya Port | 4.32E-01 | 1.24E-01 | 1.55E-02 | 2.21E-02 | 1.20E+01 | 3.45E+00 | 4.32E-01 | 6.14E-01 | - | - | - | - |
| 7 | Kumyaka | 5.62E+00 | 1.62E+00 | 2.02E-01 | 2.88E-01 | 3.40E+01 | 9.76E+00 | 1.22E+00 | 1.74E+00 | - | - | - | - |
|  | |  |  |  |  |  |  |  |  |  |  |  |  |
| Metals | | Cr | | | | Ni | | | | Mn | | | |
| Groups | | C | A | WA | MA | C | A | WA | MA | C | A | WA | MA |
| No | Stations |  |  |  |  |  |  |  |  |  |  |  |  |
| 1 | Armutlu | 3.71E-01 | 1.07E-01 | 1.33E-02 | 1.90E-02 | 5.49E-01 | 1.58E-01 | 1.97E-02 | 2.81E-02 | 1.90E+00 | 5.46E-01 | 6.83E-02 | 9.71E-02 |
| 2 | Narlı | - | - | - | - | - | - | - | - | 5.47E-01 | 1.57E-01 | 1.97E-02 | 2.80E-02 |
| 3 | Küçükkumla | 3.01E-01 | 8.66E-02 | 1.08E-02 | 1.54E-02 | 3.47E-01 | 9.97E-02 | 1.25E-02 | 1.77E-02 | 1.73E+00 | 4.98E-01 | 6.24E-02 | 8.87E-02 |
| 4 | Gemlik Port | 2.88E-01 | 8.28E-02 | 1.04E-02 | 1.47E-02 | 4.83E-01 | 1.39E-01 | 1.74E-02 | 2.47E-02 | 1.46E+00 | 4.20E-01 | 5.25E-02 | 7.46E-02 |
| 5 | Kurşunlu Pier | 4.60E-01 | 1.32E-01 | 1.66E-02 | 2.35E-02 | 5.61E-01 | 1.61E-01 | 2.02E-02 | 2.87E-02 | 1.99E+00 | 5.72E-01 | 7.15E-02 | 1.02E-01 |
| 6 | Mudanya Port | - | - | - | - | 2.70E-01 | 7.76E-02 | 9.72E-03 | 1.38E-02 | 6.68E-01 | 1.92E-01 | 2.40E-02 | 3.42E-02 |
| 7 | Kumyaka | 2.57E-01 | 7.40E-02 | 9.26E-03 | 1.32E-02 | 4.61E-01 | 1.33E-01 | 1.66E-02 | 2.36E-02 | 7.81E-01 | 2.24E-01 | 2.81E-02 | 3.99E-02 |

C: Child, A: Adolescent, WA: Woman adult, MA: Man adult, “-“: not calculated due to below detection limit of elements

Table S8: THQ and HI values for mussels

| St No | Risk group | **Target Hazard Quotient (THQ)** | | | | | | **HI** |
| --- | --- | --- | --- | --- | --- | --- | --- | --- |
|  |  | Cu | Zn | Co | Cr | Ni | Mn |  |
| 1 | Child | 3.07E-01 | 3.53E+01 | 1.07E+00 | 1.76E+01 | 3.92E+00 | 1.94E+00 | 60.19 |
|  | Adolescent | 8.83E-02 | 1.01E+01 | 3.08E-01 | 5.07E+00 | 1.13E+00 | 5.57E-01 | 17.30 |
|  | Woman | 1.11E-02 | 1.27E+00 | 3.86E-02 | 6.35E-01 | 1.41E-01 | 6.97E-02 | 2.17 |
|  | Man | 1.57E-02 | 1.81E+00 | 5.49E-02 | 9.03E-01 | 2.00E-01 | 9.90E-02 | 3.08 |
| 2 | Child | 1.17E+00 | 7.16E+00 | - | - | - | 5.58E-01 | 8.89 |
|  | Adolescent | 3.35E-01 | 2.06E+00 | - | - | - | 1.60E-01 | 2.55 |
|  | Woman | 4.20E-02 | 2.58E-01 | - | - | - | 2.01E-02 | 0.32 |
|  | Man | 5.96E-02 | 3.66E-01 | - | - | - | 2.85E-02 | 0.45 |
| 3 | Child | 1.38E-01 | 1.30E+01 | - | 1.44E+01 | 2.48E+00 | 1.77E+00 | 31.73 |
|  | Adolescent | 3.96E-02 | 3.74E+00 | - | 4.13E+00 | 7.12E-01 | 5.09E-01 | 9.12 |
|  | Woman | 4.96E-03 | 4.68E-01 | - | 5.16E-01 | 8.91E-02 | 6.37E-02 | 1.14 |
|  | Man | 7.05E-03 | 6.64E-01 | - | 7.34E-01 | 1.27E-01 | 9.05E-02 | 1.62 |
| 4 | Child | 1.85E-01 | 1.58E+01 | - | 1.37E+01 | 3.45E+00 | 1.49E+00 | 34.65 |
|  | Adolescent | 5.32E-02 | 4.54E+00 | - | 3.94E+00 | 9.92E-01 | 4.28E-01 | 9.96 |
|  | Woman | 6.66E-03 | 5.69E-01 | - | 4.93E-01 | 1.24E-01 | 5.36E-02 | 1.25 |
|  | Man | 9.46E-03 | 8.08E-01 | - | 7.01E-01 | 1.77E-01 | 7.62E-02 | 1.77 |
| 5 | Child | 2.61E-01 | 2.18E+01 | 8.17E-01 | 2.19E+01 | 4.01E+00 | 2.03E+00 | 50.79 |
|  | Adolescent | 7.49E-02 | 6.26E+00 | 2.35E-01 | 6.30E+00 | 1.15E+00 | 5.83E-01 | 14.60 |
|  | Woman | 9.38E-03 | 7.83E-01 | 2.94E-02 | 7.88E-01 | 1.44E-01 | 7.30E-02 | 1.83 |
|  | Man | 1.33E-02 | 1.11E+00 | 4.18E-02 | 1.12E+00 | 2.05E-01 | 1.04E-01 | 2.60 |
| 6 | Child | 1.23E-01 | 5.72E+00 | - | - | 1.93E+00 | 6.82E-01 | 8.46 |
|  | Adolescent | 3.55E-02 | 1.64E+00 | - | - | 5.55E-01 | 1.96E-01 | 2.43 |
|  | Woman | 4.44E-03 | 2.06E-01 | - | - | 6.94E-02 | 2.45E-02 | 0.30 |
|  | Man | 6.31E-03 | 2.93E-01 | - | - | 9.86E-02 | 3.49E-02 | 0.43 |
| 7 | Child | 1.61E+00 | 1.62E+01 | - | 1.23E+01 | 3.29E+00 | 7.96E-01 | 34.12 |
|  | Adolescent | 4.62E-01 | 4.65E+00 | - | 3.52E+00 | 9.47E-01 | 2.29E-01 | 9.81 |
|  | Woman | 5.78E-02 | 5.82E-01 | - | 4.41E-01 | 1.19E-01 | 2.87E-02 | 1.23 |
|  | Man | 8.21E-02 | 8.27E-01 | - | 6.27E-01 | 1.68E-01 | 4.07E-02 | 1.75 |
